# Assessing factors associated with poor maternal mental health among mothers of children born small and sick at 24–47 months in rural Rwanda

**DOI:** 10.1186/s12884-020-03301-3

**Published:** 2020-10-21

**Authors:** Marie Claire Abimana, Egide Karangwa, Ibrahim Hakizimana, Catherine M. Kirk, Kathryn Beck, Ann C. Miller, Silas Havugarurema, Sadallah Bahizi, Alphonsine Uwamahoro, Kim Wilson, Mathieu Nemerimana, Alphonse Nshimyiryo

**Affiliations:** 1 Partners In Health/Inshuti Mu Buzima, Rwinkwavu, Rwanda; 2 Division of Global Health and Social Medicine, Harvard Medical School, Boston, USA; 3 Rwanda Ministry of Health, Kirehe District Hospital, Kirehe, Rwanda; 4 Division of General Pediatrics, Boston Children’s Hospital, Boston, USA

**Keywords:** Maternal mental health, Prematurity, Low birthweight, Rwanda, Nurturing care, Toxic stress, Developmental disability

## Abstract

**Background:**

Global investments in neonatal survival have resulted in a growing number of children with morbidities surviving and requiring ongoing care. Little is known about the caregivers of these children in low- and middle-income countries, including maternal mental health which can further negatively impact child health and development outcomes. We aimed to assess the prevalence and factors associated with poor maternal mental health in mothers of children born preterm, low birthweight (LBW), and with hypoxic ischemic encephalopathy (HIE) at 24–47 months of age in rural Rwanda.

**Methods:**

Cross-sectional study of children 24–47 months born preterm, LBW, or with HIE, and their mothers discharged from the Neonatal Care Unit (NCU) at Kirehe Hospital between May 2015–April 2016 or discharged and enrolled in a NCU follow-up program from May 2016–November 2017. Households were interviewed between October 2018 and June 2019. Mothers reported on their mental health and their child’s development; children’s anthropometrics were measured directly. Backwards stepwise procedures were used to assess factors associated with poor maternal mental health using logistic regression.

**Results:**

Of 287 total children, 189 (65.9%) were born preterm/LBW and 34.1% had HIE and 213 (74.2%) screened positive for potential caregiver-reported disability. Half (*n* = 148, 51.6%) of mothers reported poor mental health. In the final model, poor maternal mental health was significantly associated with use of violent discipline (Odds Ratio [OR] 2.29, 95% Confidence Interval [CI] 1.17,4.45) and having a child with caregiver-reported disability (OR 2.96, 95% CI 1.55, 5.67). Greater household food security (OR 0.80, 95% CI 0.70–0.92) and being married (OR = 0.12, 95% CI 0.04–0.36) or living together as if married (OR = 0.13, 95% CI 0.05, 0.37) reduced the odds of poor mental health.

**Conclusions:**

Half of mothers of children born preterm, LBW and with HIE had poor mental health indicating a need for interventions to identify and address maternal mental health in this population. Mother’s poor mental health was also associated with negative parenting practices. Specific interventions targeting mothers of children with disabilities, single mothers, and food insecure households could be additionally beneficial given their strong association with poor maternal mental health.

## Background

Globally, over 20 million children are born at low birthweight (LBW < 2500 g) each year, nearly 15 million children are born preterm (gestational age < 37 weeks), 32 million infants are born small for their gestational age, and 1 million newborns have neonatal encephalopathy due to birth complications [1–4]. A third of neonatal deaths are due to complications of prematurity and a quarter are due to intrapartum complications such as birth asphyxia which leads to hypoxic ischemic encephalopathy (HIE) [5]. Children born preterm, LBW or with HIE and other medical vulnerabilities also have an increased risk for poor physical and psychosocial developmental outcomes [6, 7]. In turn, mothers of these children are vulnerable to psychological distress from caring responsibilities and ongoing challenges such as poor growth that many of these children may experience [8–11]. Studies that compared parenting stress between mothers of preterm and term babies revealed that having a preterm child is associated with high levels of depressive symptoms and stress [9, 12–14].

Understanding the burden of poor mental health on mothers of children born small and sick is essential to inform interventions that promote the well-being of parents and better developmental outcomes of their children. While effects of maternal stress and preterm birth outcomes may vary by context, poor caregiver’s mental health is associated with poor infant growth and cognitive development [13, 15–19]. However, treatment approaches based on increasing social support and enhancing mother–infant interactions have been proven effective to improve maternal mental health status and simultaneously improve child developmental status [10, 20].

Despite the effectiveness of treatment, in most low- and middle-income countries, maternal mental health services have not been incorporated into the primary health care system [21], and 76–85% patients with mental health problems do not receive any intervention [19]. In Africa and Asia, the prevalence of maternal depression is estimated at 15–28% [10]. Additionally, in a post-conflict country, such as Rwanda, there is an increased likelihood of maternal distress disorders [22] which may be further exacerbated in cases of prematurity, LBW and HIE. Poor maternal mental health has been identified among caregivers of children born small or with HIE in high-income countries, in part due to the stresses of uncertain survival and potential ongoing impairment among children with these conditions [23, 24]. However, despite some small studies identifying a high prevalence of maternal depression and anxiety in areas of Rwanda [25, 26], the country’s decentralized mental health services are focused on more severe mental illnesses, such as schizophrenia [27]. Like many other low- and middle-income countries there are no specific interventions for maternal depressive symptoms and anxiety [28]. Thus, maternal depression and other distress disorders which are frequently complicated by social, emotional, and financial difficulties [29], go unaddressed. The existing evidence suggests that interventions to improve maternal mental health would also contribute to reducing the burden of childhood undernutrition so would be mutually beneficial to mother and child [30].

Rwanda has made efforts in improving outcomes for children in early childhood through improved health services at health facilities, community-based interventions for children under 5 years including community case management and community nutritional screening, as well as a growing priority on promoting optimal early childhood development [31, 32]. In Rwanda, 10% of newborns are born preterm each year and 7% are born with LBW [33]. Prematurity/LBW and HIE are two of the three leading causes of admission to hospital neonatal care units in Rwanda [34]. However, to our knowledge, mental health among mothers of children with these perinatal risk factors has not been studied in Rwanda. Therefore, this study aims to assess the prevalence and factors associated with poor maternal mental health among mothers of children born preterm, LBW or with HIE when the children are ages 24–47 months.

## Methods

### Study setting

This study was conducted in Kirehe District in the Eastern Province of Rwanda. Kirehe District is served by Kirehe District Hospital (KDH), which is operated by the Rwanda Ministry of Health (MOH) and serves a catchment of over 400,000 people [35] plus a refugee camp of 60,000 people [36]. Kirehe District Hospital has been supported by Partners In Health/Inshuti Mu Buzima since 2008. The hospital has provided specialized inpatient neonatal care for preterm and LBW newborns, as well as newborns with HIE and other medical conditions since 2012. In May 2016, a Pediatric Development Clinic (PDC) was established at Kirehe District Hospital through the collaboration of Partners In Health/Inshuti Mu Buzima and the MOH to provide clinical, nutritional, and developmental follow-up to children discharged from the Kirehe District Hospital neonatol care unit (NCU). Detailed information on PDC can be found elsewhere [37].

### Study design and population

This was a cross-sectional study that included children aged 24–47 months born with prematurity, LBW, or HIE, and discharged from the NCU at Kirehe District Hospital between May 2015 and April 2016 or enrolled in the PDC from May 2016 to November 2017. Primary caregivers of these children were included in this study if they were the biological mothers and if data on their mental health status was collected.

### Data collection

Data were collected between October 2018 and June 2019 by a trained team of Partners In Health/Inshuti Mu Buzima data collectors. Data collectors conducted household interviews in Kinyarwanda with primary caregivers of eligible children, and measured anthropometrics and developmental indicators for children. Due to limitations in literacy, the data collector conducted the consent process and interview verbally and written confirmation of consent was provided by participants with a signature or fingerprint. The collected data included caregiver-reported child’s developmental status, child’s caregiver-reported disability status, child feeding, caregiver mental health, household socio-economic status, child discipline practices, and early stimulation activities. The child’s nutritional status was measured directly.

#### Measures

##### Primary outcome

Maternal mental health was measured using the Hopkins Symptom Checklist, which has been validated in Rwanda. It is a 25 item measure of symptoms of anxiety and depression with the total score being a mean of all items; a score > 1.75 is considered high level of anxiety and depression symptoms [38]. The full questionnaire is available in Supplementary File 1.

##### Covariates

Stunting (low height-for-age), wasting (low weight-for-height), and underweight (low weight-for-age) were measured using standard anthropometric procedures to capture weight and height (or length) and compared to the WHO Child Growth Standards [39]. The cutoff point for severe malnutrition was a z-score less than − 3 standard deviations and moderate malnutrition is a z-score less than − 2 standard deviations using the WHO Child Growth Standards.

Home environment was assessed using UNICEF’s Family Care Indicators [40], which measure early learning activities, availability of play materials, and exposure to inadequate care. Inadequate care is when caregivers reported that the child was left alone, or the child was cared for by another child less than 10 years of age, for 1 hour or more in the past week. The UNICEF Multiple Indicator Cluster Survey measure of violent discipline [41] was also used. Exposure to violent discipline was defined as caregiver-reported use of any psychological aggression (i.e., yelling, name calling) or physical punishment (i.e., hitting, spanking) to the child in the past month.

Household socioeconomic status was measured using an asset index which was analyzed using principal component analysis for a measure of relative wealth in our sample; households were grouped into tertiles representing the poorest, the middle, and the wealthiest groups. In addition, the Rwandan “Ubudehe” categorization was collected, which is a Rwandan community-based ranking of wealth status. Households are categorized in to 4 groups with the first category being the extremely poor and qualifying for government social services including free health insurance, and the fourth being the wealthiest category.

Child disability was measured using the Ten Questions Questionnaire [42], which is a caregiver-reported screening tool for detecting impairments in physical development, hearing, vision, and communication. Children whose mother responded to yes on any one impairment were considered at risk for moderate to severe disability.

Child development was measured using the Ages and Stages Questionnaire (ASQ-3) [43]. The ASQ-3 asks 30 age-specific questions across communication, gross motor, fine motor, personal social, and problem solving domains. Children are screened as “at risk for developmental delay” if they fall below the standard Western-normed cut-point in any one of the five domains.

Social support was measured using the Inventory of Socially Supportive Behaviors which had been previously adapted to Rwanda and is scored as a mean score of all items with 1 being the minimum score (low social support) and 5 being the maximum [44, 45].

Months of Adequate Household Food Provisioning is a way to measure self-reported household food access over the past year [46].

### Data analysis

We described socio-economic and demographic characteristics, child nutrition and development, home environment and maternal mental health using frequencies and percentages for categorical variables and mean and standard deviation (SD) or median and interquartile range (IQR) for continuous variables. Bivariate associations between maternal mental health status and each covariate was assessed using Fisher’s exact (categorical) and t-test (continuous) or Wilcoxon rank sum test if continuous measures were not normally distributed (social support, months of adequate household food provisioning). Backwards stepwise techniques were used to build a multivariable logistic regression model to investigate factors associated with poor maternal mental health. We included in the full model all variables associated with maternal mental health at α = 0.20 in the bivariate analysis. Manual removal of variables was completed following backwards stepwise procedures and the final model included only variables significant at α = 0.05.

## Results

Our study included 287 children born preterm/LBW (*n* = 189, 65.9%) or with HIE (*n* = 98, 34.1%) and their biological mothers (Table 1). Of these children, 50.9% (*n* = 146) were boys and 63.1% (*n* = 181) were aged 24–35 months. There were 15.3% (*n* = 44) single-mothers and 60.3% (*n* = 173) mothers had not completed any formal education. The most common playthings available included 64.5% (*n* = 185) children who played with homemade toys and 69.0% (*n* = 198) of children who play with household objects. In parenting practices, 73.9% (*n* = 212) of children experienced any violent discipline, 43.2% (*n* = 124) had inadequate care, and 49.1% (*n* = 141) of mothers reported engaging in four or more activities to promote learning. More children with HIE were stunted (*n* = 45/98, 61.6%), underweight (*n* = 43/98, 44.7%), and wasted (*n* = 8/98, 11.0%) than children born preterm/LBW among whom 56.4% (*n* = 101/189) were stunted, 29.0% (*n* = 54/189) were underweight and 3.9% (*n* = 7/189) were wasted. Overall, 74.2% of children (*n* = 213) were at-risk for developmental delay, with 72.2% (*n* = 135/187) of preterm/LBW children and 89.7% (*n* = 85/97) of children with HIE. Potential disability was reported by the caregiver for 74.2% of children (n = 213), with 68.3% (*n* = 129/189) among preterm/LBW and 85.7% (*n* = 84/98) among children with HIE. Half of mothers (*n* = 148, 51.6%) reported poor mental health.

**Table 1:** Clinical and socio-demographic characteristics of children, mothers, and the home environment

	Total (***N*** = 287)		Preterm/LBW (***N*** = 189)		HIE (N = 98)	
	N	%	n	%	n	%
**Child’s Sex**						
Male	146	50.9%	91	48.2%	55	56.1%
Female	141	49.1%	98	51.9%	43	43.9%
**Child Age**						
24–35 Months	181	63.1%	137	72.5%	44	44.9%
36–47 Months	106	36.9%	52	27.5%	54	55.1%
**Number of People Living in the Household**						
2–3 People	65	22.7%	37	19.6%	28	28.6%
4–5 People	110	38.3%	68	36.0%	42	42.9%
6 or More	112	39.0%	84	44.4%	28	28.6%
**Eligible for Government Social Services (*n*** **= 280)**						
Yes (Ubudehe 1)	31	11.1%	18	9.8%	13	13.4%
No	249	88.9%	165	90.2%	84	86.6%
**Socioeconomic Status**						
Poorest Tertile	104	36.2%	71	37.6%	33	33.7%
Middle Tertile	89	31.0%	56	29.6%	33	33.7%
Wealthiest Tertile	94	32.8%	62	32.8%	32	32.7%
**Single or Multiple Birth**						
Single	230	80.1%	135	71.4%	95	96.9%
Twins or Triplets	57	19.9%	54	28.6%	3	3.1%
**Birthweight in Grams, mean (SD)**	2133.8	743.7	1718.1	380.6	2943.2	596.3
**Mother’s Age at Interview, mean (SD)**	31.1	6.7	31.7	6.8	29.8	6.3
**Marital Status**						
Single	44	15.3%	29	15.3%	15	15.3%
Married	142	50.0%	95	50.3%	47	48.0%
Living with Partner (not legally married)	101	35.2%	65	34.4%	36	36.7%
**Mother is Literate**						
No	78	27.2%	53	28.0%	25	25.5%
Yes	209	72.8%	136	72.0%	73	74.5%
**Mother’s Highest Level of Education Completed**						
None	173	60.3%	109	57.7%	64	65.3%
Primary school or Higher Completed	114	39.7%	80	42.3%	34	34.7%
**Child Having any Picture/Children’s Books**						
No	259	90.9%	169	89.9%	90	92.8%
Yes	26	9.1%	19	10.1%	7	7.2%
**Child Plays with Homemade Toys (such as dolls, cars, or other toys)**						
No	102	35.5%	61	32.3%	41	41.8%
Yes	185	64.5%	128	67.7%	57	58.2%
**Child Plays with Toys from a Shop or Manufactured Toys**						
No	239	83.3%	156	82.5%	83	84.7%
Yes	48	16.7%	33	17.5%	15	15.3%
**Child Plays with Household Objects (such as bowls or pots) or Objects**						
No	89	31.0%	51	27.0%	38	38.8%
Yes	198	69.0%	138	73.0%	60	61.2%
**Child Discipline**						
No Violent Discipline	75	26.1%	35	18.5%	40	40.8%
Any Violent Discipline	212	73.9%	154	81.5%	58	59.2%
**Child Experienced Any Inadequate Care**						
No	163	56.8%	107	56.6%	56	57.1%
Yes, left alone or with a child under 10 for 1+ hours	124	43.2%	82	43.4%	42	42.9%
**Mother Engaged in 4+ Activities to Support Learning**						
No	146	50.9%	93	49.2%	53	54.1%
Yes	141	49.1%	96	50.8%	45	45.9%
**Mother-Reported Social Support, median (IQR)**	2.35	1.97, 2.77	2.38	1.96, 2.83	2.27	2.00, 2.74
**Months of Adequate Household Food Provision in Last Year, median (IQR)**	9	7, 10	9	7, 10	9	7, 10
**Minimum Dietary Diversity: Child ate from 4+ IYCF Food Groups**						
No	110	38.3%	75	39.7%	35	35.7%
Yes	177	61.7%	114	60.3%	63	64.3%
**Height-for-Age (Stunting),** ***n*** **= 252**						
Normal	106	42.1%	78	43.6%	28	38.4%
Stunted	146	57.9%	101	56.4%	45	61.6%
**Weight-for-Age (Underweight),** ***n*** **= 284**						
Normal	187	65.9%	132	70.7%	55	56.1%
Underweight	97	34.2%	54	29.0%	43	44.7%
**Weight-for-Height (Wasting),** ***n*** **= 254**						
Normal	239	94.1%	174	96.1%	65	89.0%
Wasted	15	5.9%	7	3.9%	8	11.0%
**Child Development on ASQ-3, n = 284**						
On-Track	62	21.8%	52	27.8%	10	10.3%
At Risk for Developmental Delay	222	78.2%	135	72.2%	87	89.7%
**Caregiver-reported Child Disability**						
No	74	25.8%	60	31.8%	14	14.3%
Potential Disability	213	74.2%	129	68.3%	84	85.7%
**Maternal Mental Health**						
Normal	139	48.4%	92	48.7%	47	48.0%
Poor	148	51.6%	97	51.3%	51	52.0%

In the bivariate analysis (Table 2), at α < 0.20, maternal mental health status was associated with the child’s sex (*p* = 0.195), age of the child (*p* = 0.050), household size (*p* = 0.077), caregiver’s eligibility status for government social services (*p* = 0.057), socio-economic status (*p* = 0.168), mother’s age (*p* = 0.030), marital status (*p* = 0.003), mother’s literacy (*p* = 0.002), mother’s level of education (*p* = 0.040), child having any picture/children’s book (*p* = 0.098), child discipline (*p* = 0.011), child experiencing any inadequate care (*p* = 0.001), mother-reported social support (*p* = < 0.001), months of adequate household food provision in a year (*p* < 0.001), child’s access to the minimum dietary diversity (*p* = 0.052), child underweight status (*p* = 0.017), child wasting status (*p* = 0.067), and child disability as reported by the caregiver (*p* = 0.003).

**Table 2:** Bivariate associations between child and household factors and maternal mental health status

Variable	Normal Mental Health		Poor Mental Health		***p***-value
	n	%	n	%	
**Child’s Sex**					
Male	65	46.8%	81	54.7%	0.195
Female	74	53.2%	67	45.3%	
**Child Age**					
24–35 Months	96	69.1%	85	57.4%	0.050
36–47 Months	43	30.9%	63	42.6%	
**Child’s Condition**					
Preterm/LBW	92	66.2%	97	65.5%	> 0.999
HIE	47	33.8%	51	34.5%	
**Number of People Living in the Household**					
2–3 People	36	25.9%	29	19.6%	0.077
4–5 People	58	41.7%	52	35.1%	
6 or More	45	32.4%	67	45.3%	
**Eligible for Government Social Services (n = 280)**					
Yes (Ubudehe 1)	10	7.3%	21	14.7%	0.057
No	127	92.7%	122	85.3%	
**Socioeconomic Status**					
Poorest Tertile	47	33.8%	57	38.5%	0.168
Middle Tertile	39	28.1%	50	33.8%	
Wealthiest Tertile	53	38.1%	41	27.7%	
**Single or Multiple Birth**					
Single	112	80.6%	118	79.7%	0.883
Twins or Triplets	27	19.4%	30	20.3%	
**Birthweight in Grams, mean (SD)**	2080.8	721.8	2181.5	762.1	0.261
**Mother’s Age at Interview, mean (SD)**	30.2	6.5	31.9	6.7	0.030
**Marital Status**					
Single	11	7.9%	33	22.3%	0.003
Married	74	53.2%	68	46.0%	
Living with Partner (not legally married)	54	38.9%	47	31.8%	
**Mother is Literate**					
No	25	18.7%	52	35.1%	0.002
Yes	113	81.3%	96	64.9%	
**Mother’s Highest Level of Education Completed**					
None	75	54.0%	98	66.2%	0.040
Primary school or Higher Completed	64	46.0%	50	33.8%	
**Child Having any Picture/Children’s Books**					
No	120	87.6%	139	93.9%	0.098
Yes	17	12.4%	9	6.1%	
**Child Plays with Homemade Toys (such as dolls, cars, or other toys)**					
No	47	33.8%	55	37.2%	0.622
Yes	92	66.2%	93	62.8%	
**Child Plays with Toys from a Shop or Manufactured Toys**					
No	112	80.6%	127	85.8%	0.269
Yes	27	19.4%	21	14.2%	
**Child Plays with Household Objects (such as bowls or pots) or Objects**					
No	38	27.3%	51	34.5%	0.204
Yes	101	72.7%	97	65.5%	
**Child Discipline**					
No Violent Discipline	46	33.1%	29	19.6%	0.011
Any Violent Discipline	93	66.9%	119	80.4%	
**Child Experienced Any Inadequate Care**					
No	93	66.9%	70	47.3%	0.001
Yes, left alone or with a child under 10 for 1h hours	46	33.1%	78	52.7%	
**Mother Engaged in 4+ Activities to Support Learning**					
No	69	49.6%	77	52.0%	0.724
Yes	70	50.4%	71	48.0%	
**Mother-Reported Social Support, median (IQR)**	2.6	2.16, 2.97	2.2	1.84, 2.65	< 0.001
**Months of Adequate Household Food Provision in Last Year, median (IQR)**	9.0	8, 10	8.0	6,10	< 0.001
**Minimum Dietary Diversity: Child Ate from 4+ IYCF Food Groups**					
No	45	32.4%	65	43.9%	0.052
Yes	94	67.6%	83	56.1%	
**Height-for-Age (Stunting), n = 252**					
Normal	53	42.1%	53	42.1%	> 0.999
Stunted	73	57.9%	73	57.9%	
**Weight-for-Age (Underweight), n = 284**					
Normal	100	73.0%	87	59.2%	0.017
Underweight	37	27.0%	60	40.8%	
**Weight-for-Height (Wasting), n = 254**					
Normal	124	96.9%	115	91.3%	0.067
Wasted	4	3.1%	11	8.7%	
**Child Development on ASQ-3**					
On-Track	32	23.2%	30	20.6%	0.668
At Risk for Developmental Delay	106	76.8%	116	79.5%	
**Caregiver-reported Child Disability**					
No	47	33.8%	27	18.2%	0.003
Potential Disability	92	66.2%	121	81.8%	

Factors associated with increased odds of poor maternal mental health in the final model included caregiver-reported child disability (Adjusted odds Ratio [AOR]: 2.96; 95% Confidence Interval [CI]: 1.55–5.67) and child exposure to violent discipline (AOR: 2.29; 95%CI: 1.17–4.45) (Table 3). The odds of poor maternal mental health were significantly lower among mothers living with their partners in either legal marriage (AOR: 0.12; 95%CI: 0.04–0.36) or cohabitating (AOR: 0.13; 95%CI: 0.05–0.37) compared to single mothers, as well as increased number of months of adequate household food provision (AOR: 0.80; 95%CI: 0.70–0.92).

**Table 3:** Multivariable logistic regression predicting poor maternal mental health

	Full Model			Final Model		
Variable	AOR	95% CI	***p***-value	AOR	95% CI	***p***-value
**Child’s Sex**						
Male	ref					
Female	1.06	0.55, 2.04	0.864			
**Child Age**						
24–35 Months	ref					
36–47 Months	2.08	1.01, 4.30	0.048			
**Number of People Living in the Household**						
2–3 People	ref					
4–5 People	0.80	0.31, 2.03	0.635			
6 or more	1.46	0.47, 4.56	0.517			
**Eligible for Government Social Services (*n*** **= 269)**						
No	ref					
Yes (Ubudehe 1)	1.87	0.49, 7.18	0.363			
**Single or Multiple Birth**						
Single	ref					
Twins or Triplets	0.77	0.35, 1.69	0.509			
**Marital Status**						
Single	ref			ref		
Married	0.20	0.56, 0.74	0.015	0.12	0.04, 0.36	< 0.001
Living with Partner (not legally married)	0.28	0.08, 1.01	0.051	0.13	0.05, 0.37	< 0.001
**Mother is Literate**						
No	ref					
Yes	0.64	0.28, 1.47	0.298			
**Mother’s Highest Level of Education Completed**						
None	ref					
Primary school or Higher Completed	0.86	0.40, 1.84	0.694			
**Child Having any Picture/Children’s books**						
No	ref					
Yes	0.78	0.27, 2.27	0.649			
**Child Discipline**						
No Violent Discipline	ref			ref		
Any Violent Discipline	4.83	1.90, 12.28	0.001	2.29	1.17, 4.45	0.015
**Child Experienced Any Inadequate Care**						
No	ref					
Yes	1.49	0.76, 2.92	0.246			
**Mean Mother-Reported Social Support, median (IQR)**	0.59	0.31, 1.13	0.110			
**Months of Adequate Household Food Provision in Last Year, median (IQR)**	0.89	0.74, 1.06	0.186	0.80	0.70, 0.92	0.002
**Weight-for-Age (Underweight)**						
Normal	ref					
Underweight	1.48	0.63, 3.47	0.364			
**Weight-for-Height (Wasting)**						
Normal	ref					
Wasted	6.92	0.94, 51.21	0.058			
**Caregiver-reported Child Disability**						
No	ref			ref		
Potential Disability	2.47	1.15, 5.30	0.02	2.96	1.55, 5.67	0.001

Full and final model are controlled for mother’s age and socioeconomic status. AOR (adjusted odds ratio)

## Discussion

In this study, we found a high burden of poor maternal mental health (51.6%) among mothers with children born preterm, LBW and/or with HIE when the children were ages 24–47 months. This poor mental health was significantly associated with having a child with a caregiver-reported disability, single marital status, food insecurity in the household, and poor child discipline practices.

While the prevalence of poor maternal mental health in the population of Rwanda is unknown, we found about double the prevalence of poor maternal mental health in mothers of children born preterm/LBW or with HIE as compared to the general population of mothers in other low- and middle-income countries where the prevalence of maternal depression is estimated at 19–25% [47]. This finding is among the highest ranges in studies of poor maternal mental health from low- and middle-income countries (4.9–59.4%) [19].

Three-quarters of the children in our study screened positive for potential caregiver-reported disability and odds of poor mental health were more than double among mothers caring for children with a caregiver-reported disability. Similar findings were noted in Malawi where 41.2% of caregivers of children with disability, including preterm/LBW and HIE children, reported having psychological distress [48]. Caring for a child with disability can increase stress in a caregiving role, increasing risk for poor mental health. Maternal depression and negative mother-infant interactions can be exacerbated when depressed mothers perceive their infants as temperamentally difficult [10]. This finding highlights the need to provide routine mental health screening and interventions to support mothers of children with developmental difficulties and disability.

In this study, poor mental health was also found to be strongly associated with single marital status. Single mothers’ poor mental health prevalence was three times higher than married or cohabitating women. This finding is similar to what was found by other studies that the presence of a partner in the family is associated with greater life satisfaction and better maternal mental health outcomes [49]. This finding may suggest the benefit of having a partner present in the family who is socially, economically and psychologically supportive of the caregiving role of the mother. Being married has been shown to lead to reduced stress in caregiving roles and thus resulting in better maternal mental health outcomes [50].

Mothers with poor mental health were more likely to experience more months of adequate household food provision, a measure of household food access. This link between stress of insufficient food and poor maternal mental health has been well demonstrated in high-income setting [51], with detriment to children’s outcomes [52]. Household food insecurity in our sample was found along with a high prevalence of malnutrition and developmental delays. The burden of food insecurity is likely affecting children’s outcomes both through the pathway of increased maternal stress and poor mental health, but also directly because lack of adequate nutrition impedes children’s development [53]. Sufficient intake of micronutrients and dietary diversity is essential for optimizing children’s brain development and preventing malnutrition including anemia [54].

Poor parenting practices were associated with higher odds of poor maternal mental health. In our sample, three fourths of children were exposed to violent discipline and nearly half were exposed to inadequate care with rates of both negative parenting practices significantly higher among mothers with poor mental health status. The overall high rates of harsh disciplinary practices for all children in our study were not surprising, as these practices have been shown to be common in Rwanda and other sub-Saharan African countries [55]. This higher rate among mothers with poor mental health is supported by other studies demonstrating the increased risk of child abuse and neglect among mothers with mental health problems [56]. Further, children with caregiver-reported disability – of whom there were many in our sample – may experience violence more than non-disabled children from the time they are born [57]. Additionally, both poor maternal mental health and negative mother infant interactions can be exacerbated when a mother is perceiving her infant as having a disability [10]. These forms of compounded adversity are known to be detrimental to children’s development. Holistic, integrated programs addressing maternal mental health, social needs for caregivers of children with developmental difficulties, and children’s health, nutrition and developmental services are needed to optimize nurturing care among at-risk children. These services should be targeted to families with high-risk for poor outcomes, especially children born preterm/LBW and with HIE.

Our study has a few limitations. First, the sample size was small and from only one rural Rwandan district so our findings may not be generalizable to other regions. However, Kirehe district is a rural district with similar characteristics to other rural Rwandan districts. Child disability was reported by caregivers, and no diagnostic tool was used to confirm the child’s disability. However, we used a standard tool and our study was limited to biological mothers who are the primary caregivers to their child and are able to identify perceived disability of their child. In addition, as we are looking only at a cross-sectional study when children were ages 24–47 months, we have a period prevalence for poor maternal health but do not know the timing of onset or duration of poor mental health which means we cannot know what is a cause of versus a result of poor maternal mental health.

## Conclusions

More than half of caregivers of children born preterm, LBW and with HIE had poor mental health status, and the children had high rates of developmental delay and a caregiver-reported disability at ages 2–3 years. Services for children with perinatal risk factors must include provision of mental health screening and treatment for mothers while simultaneously addressing early intervention and care for the child’s health, nutrition, and development.

## Supplementary information

**Additional file 1.**

## Data Availability

The datasets used and/or analyzed during the current study are available from the corresponding author on reasonable request.

## Abbreviations

ASQ-3: Ages and Stages Questionnaire-3
HIE: Hypoxic Ischemic Encephalopathy
LBW: Low Birthweight
MOH: Ministry of Health
NCU: Neonatal Care Unit
PDC: Pediatric Development Clinic
WHO: World Health Organization
