# Supplementary material for: Assessing factors associated with poor maternal mental health among mothers of children born small and sick at 24–47 months in rural Rwanda
Source: BMC Pregnancy Childbirth. 2020 Oct 21;20:643. doi: 10.1186/s12884-020-03301-3 (PMC7579859; doi:10.1186/s12884-020-03301-3)
Supplement: Supplementary file 1 — Additional file 1. [file 12884_2020_3301_MOESM1_ESM.pdf]

Kirehe-PDC-FU

Field	Question	Answer
Child's Information		
child_sex	Select the sex of the child<br>*Enter only if the sex of the child from EMR needs correction.*	0 Male<br>1 Female
mult_birth *(required)*	Was the child a single or multiple birth (e.g., twins or triplets)?	1 Single birth<br>2 Twins<br>3 Triplets<br>99 Don't Know
bday *(required)*	What is {child_name}'s birthday?<br>*Verify the date from the birth registration if it was avialable. Or mutuelle/immunization card if available.*	
Caregiver Information		
cg_sex *(required)*	Enter the caregiver's sex	0 Male<br>1 Female
rel *(required)*	What is your relationship to the child?	1 Biological mother<br>2 Biological father<br>3 Adoptive mother<br>4 Adoptive father<br>5 Step-mother<br>6 Step-father<br>7 Aunt/Uncle<br>8 Grandparent<br>9 Other
rel_other *(required)*	If other, describe	
age *(required)*	How old did you turn on your last birthday?	
Information about the Caregiver		
Ed1 *(required)*	Have you ever attended school?	0 No<br>1 Yes<br>99 Don't Know
Ed2 *(required)*	What is the highest level you completed in school?	0 None<br>1 P1<br>2 P2<br>3 P3<br>4 P4<br>5 P5<br>6 P6<br>16 P7<br>17 P8<br>18 P9<br>7 S1<br>8 S2<br>9 S3<br>10 S4<br>11 S5<br>12 S6<br>13 Some college/University<br>14 College/University Degree<br>15 Seminary/Religious school only<br>20 Other
Ed2_other *(required)*	If other, describe	
Ed5 *(required)*	Are you able to read and write?	0 No<br>1 Yes<br>99 Don't Know
Mar1 *(required)*	Are you currently married or living together with a partner as if married?	1 Yes, Currently married<br>2 Yes, Currently living with a partner<br>0 No, Not in a union<br>99 I don't know
Mar2 *(required)*	Is your partner living with you now or is he/she staying elsewhere?	1 Yes, living with parther<br>0 No, staying elsewhere<br>99 I don't know
Family Composition		
hh_size *(required)*	In total, how many people live in your household?	
hh_u17 *(required)*	Including the index child ({child_name}),, how many children 17 years old or younger live in your household?	
hh_u5 *(required)*	Including the index child ({child_name}), how many children under 5, who usually live in your home, are in the household?	

Field	Question	Answer
father_edu *(required)*	What is the highest level the father of ({child_name}) completed in school?	0 None<br>1 P1<br>2 P2<br>3 P3<br>4 P4<br>5 P5<br>6 P6<br>16 P7<br>17 P8<br>18 P9<br>7 S1<br>8 S2<br>9 S3<br>10 S4<br>11 S5<br>12 S6<br>13 Some college/University<br>14 College/University Degree<br>15 Seminary/Religious school only<br>20 Other
father_home *(required)*	Do you live together with the biological father of ({child_name})?	0 No<br>1 Yes<br>99 Don't Know
father_age *(required)*	How old did the child's father turn on his last birthday?<br>*Enter 999 if don't know*	
home4 *(required)*	Does the father spend some time every day caring for the child? For example talking, walking, and/or playing with the child?<br>*In a home setting in which the mother is the primary caregiver, interactions with the father, or father figure such as a grandfather, uncle, step-father, etc. living in the home, allow the child to experience variety in social, emotional, and play interactions. Score "Yes" if the father regularly spends time with the child. Score "No" if this never or rarely happens, even if this is because the father is often away for work or other reasons.*	0 No<br>1 Yes<br>99 Don't Know
mother_edu *(required)*	What is the highest level the mother of ({child_name}) completed in school?	0 None<br>1 P1<br>2 P2<br>3 P3<br>4 P4<br>5 P5<br>6 P6<br>16 P7<br>17 P8<br>18 P9<br>7 S1<br>8 S2<br>9 S3<br>10 S4<br>11 S5<br>12 S6<br>13 Some college/University<br>14 College/University Degree<br>15 Seminary/Religious school only<br>20 Other
mother_home *(required)*	Do you live together with the biological mother of ({child_name})?	0 No<br>1 Yes<br>99 Don't Know
mother_age *(required)*	How old did the child's mother turn on his last birthday?<br>*Enter 999 if don't know*	
PDC Feedback		
pdc_current *(required)*	Do you still take your child to the PDC for scheduled visits?<br>-	0 No<br>1 Yes<br>99 Don't Know
pdc_stop *(required)*	Why do you not take your child to the PDC any more?<br>-	

Field	Question	Answer
pdc1 *(required)*	What did you like about the PDC?	0 Nothing<br>1 PDC helped my child to be healthier<br>2 PDC connected me to mothers with similar children<br>3 PDC providers were helpful<br>4 I learned how to care for my child in PDC<br>5 PDC provided food for my family<br>6 PDC paid for transport<br>20 Other
pdc1_other *(required)*	Other, describe:	
pdc2 *(required)*	How would you rate the quality of the services you received in PDC?	4 Excellent<br>3 Good<br>2 Average<br>1 Below average<br>0 Poor<br>99 Don't Know
pdc3 *(required)*	What challenges did you experience accessing the PDC program?	1 The waiting time is too long<br>2 There are other children who I can not leave unattended at home<br>3 Do not see progress in my child's condition<br>4 My partner did not see value in the service<br>5 Services are too expensive<br>6 Services are of poor quality<br>7 Stigma/discrimination (would not feel that they are welcome)<br>8 Because the child is too young<br>9 Because the services are too far away<br>10 My child's condition will never get better<br>11 My child is healthy and doesn't need to go to the health center<br>12 I don't see value in the PDC program for my child<br>20 Other
pdc3_other *(required)*	Other, describe:	
pdc4 *(required)*	How satisfied are you with the PDC program?	0 Satisfied<br>1 Neither Satisfied or Dissatisfied<br>2 Dissatisfied<br>99 Don't Know
pdc5 *(required)*	Would you recommend PDC to other parents or children?	0 No<br>1 Yes<br>99 Don't Know
pdc6 *(required)*	If yes, why would you recommend it?	0 Nothing<br>1 PDC helped my child to be healthier<br>2 PDC connected me to mothers with similar children<br>3 PDC providers were helpful<br>4 I learned how to care for my child in PDC<br>5 PDC provided food for my family<br>6 PDC paid for transport<br>20 Other
pdc6_other *(required)*	Other, describe:	
Household Assets		

Field	Question	Answer
Ubu *(required)*	What is your official ubudehe status?	1 Ubudehe 1<br>2 Ubudehe 2<br>3 Ubudehe 3<br>4 Ubudehe 4<br>7 Not categorized<br>99 Don't Know<br>88 Refused to answer
WI5	Does your household have...	
WI6 *(required)*	Electricity?	0 No<br>1 Yes<br>99 Don't Know
WI7 *(required)*	Radio?	0 No<br>1 Yes<br>99 Don't Know
WI8 *(required)*	Mobile phone?	0 No<br>1 Yes<br>99 Don't Know
WI9 *(required)*	A bicycle?	0 No<br>1 Yes<br>99 Don't Know
WI10 *(required)*	Main material of the floor. Record observation.	11 Earth/sand<br>12 Dung<br>21 Wood planks<br>22 Palm/bamboo<br>31 Parquet or polished wood<br>32 Vinyl or asphalt strips<br>33 Ceramic tiles<br>34 Cement<br>35 Carpet<br>96 Other
WI11 *(required)*	If other, describe	
WI12 *(required)*	How many rooms in this household are used for sleeping?	
WI13 *(required)*	Does this household own any livestock, herds, other farm animals, or poultry?	0 No<br>1 Yes<br>99 Don't Know
cows *(required)*	How many cows does this household own?	
goats *(required)*	How many goats does this household own?	
sheep *(required)*	How many sheep does this household own?	
poultry *(required)*	How many chickens does this household own?	
pigs *(required)*	How many pigs does this household own?	
rabbits *(required)*	How many rabbits does this household own?	
housing *(required)*	Do you own or rent the house you are living in?	1 Home is owned by me/my partner<br>2 Home is owned by another friend/family member<br>3 Home is rented<br>6 Other
own_land *(required)*	Does your household own farming land including pastures for livestock?	0 No<br>1 Yes<br>99 Don't Know
cook_place *(required)*	Where do you do most of the cooking?	1 Inside your home<br>2 Outside your home in an enclosed area<br>3 Outside your home in an open area<br>99 Don't know
cook_sleep *(required)*	Do you, or your children, sleep in the same room you cook in?	0 No<br>1 Yes<br>99 Don't Know

Field	Question	Answer
cook_fuel *(required)*	What fuel do you use most often for cooking?	1 Gas<br>2 Electricity<br>3 Firewood<br>4 Charcoal<br>5 Kerosene<br>6 Bio-gas<br>7 Crop waste or animal dung<br>8 Other<br>99 Don't know
cook_fuel_other *(required)*	Specify other cooking fuel	
Health Insurance		
hh_insured *(required)*	Is any member of this household covered by health insurance?	0 No<br>1 Yes<br>99 Don't Know
ins_type *(required)*	What type of health insurance does your household have?	1 Mutuelle<br>2 RAMA<br>3 MMI<br>4 Private insurance<br>5 Other
ins_type_other *(required)*	Other, describe:	
all_insured *(required)*	Are ALL members of this household covered by this health insurance?	1 All household members<br>2 Some household members<br>99 Don't Know
child_insured *(required)*	Is {child_name} covered by this health insurance?	0 No<br>1 Yes<br>99 Don't Know
Household Savings		
savings1 *(required)*	Are there members of your household who saves with any formal savings institution (i.e., SACCO, commercial bank)	0 No<br>1 Yes<br>99 Don't Know
savings2 *(required)*	How much has your household deposited on average each month in SACCO or a commercial bank?<br>*Enter as 1,000s of RWF, enter "99" if she/he does not know.*	
savings3 *(required)*	Are there members of your household who saves with a tontine/community savings group?	0 No<br>1 Yes<br>99 Don't Know
savings4 *(required)*	How much has your household deposited on average each month in the savings group?<br>*Enter as 1,000s of RWF, enter "99" if she/he does not know.*	
Medical Expenses		
health_expend *(required)*	Did you spend money on medical expenses/health care In the past 3 months (money could have been paid in direct cash or credit)?	0 No<br>1 Yes<br>99 Don't Know
health_expend2 *(required)*	How much money did you spend (including credit)?<br>*ENTER "1234" if amount NOT known.*	
health_expend3 *(required)*	What costs did you pay for?	1 Consultation fees at a health faciity<br>2 Consultation fees for a CHW<br>3 Fees for services of traditional healer<br>4 Medicines<br>5 Supplies (gloves, fiche, specimen cups, etc.)<br>6 Laboratory Tests<br>7 Insurance premiums<br>8 Transport<br>10 Other<br>99 Don't Know
health_expend3a *(required)*	If other, describe	
pdc_expend *(required)*	How much money do you spend for a typical PDC visit? Include how much you spend on transport, clinic fees, medicines, or other expenses for the PDC visit.<br>*ENTER "1234" if amount NOT known.*	
Decision Making		
dm_note	In your family, who takes the final decision in the household that affects {child_name}?	

Field	Question	Answer
dm_childsick *(required)*	Decide the action when child is sick	0 Father<br>1 Mother<br>2 Father and Mother equally<br>3 Grandfather<br>4 Grandmother<br>5 Others<br>99 Don't Know
dm_childeat *(required)*	Decide what the child eats	0 Father<br>1 Mother<br>2 Father and Mother equally<br>3 Grandfather<br>4 Grandmother<br>5 Others<br>99 Don't Know
dm_money *(required)*	Decides how money is used/spent	0 Father<br>1 Mother<br>2 Father and Mother equally<br>3 Grandfather<br>4 Grandmother<br>5 Others<br>99 Don't Know
WASH		
WI1 *(required)*	What is the main source of drinking water for your household?	11 Piped into dwelling<br>12 Piped into yard/plot<br>13 Public tap/standpipe<br>21 Tube well or borehole<br>31 Protected dug well<br>32 Unprotected dug well<br>41 Protected spring<br>42 Unprotected spring<br>51 Rainwater<br>61 Tanker truck<br>71 Cart with small tank<br>81 Surface water (river/dam/lake/pond/stream/canal/irrigation channel)<br>91 Bottled water<br>96 Other
WI2 *(required)*	If other, describe	
water_insuf1 *(required)*	In the past two weeks, has there been any time when your household did not have sufficient quantities of drinking water when needed?	0 No<br>1 Yes<br>99 Don't Know
WI3 *(required)*	What kind of toilet facility does your household use?	11 Flush to piped sewer system<br>12 Flush to septic tank<br>13 Flush to pit latrine<br>14 Flush to somewhere else<br>15 Flush, don't know where<br>21 Ventilated improved pit (VIP) latrine<br>22 Pit latrine with slab<br>23 Pit latrine without slab/open pit<br>31 Composting toilet<br>41 Bucket toilet<br>51 Hanging toilet/hanging latrine<br>61 None (bush, field, stream)<br>96 Other
WI4 *(required)*	If other, describe	
WS10 *(required)*	We would like to learn about the places that households use to wash their hands.<br><br>Can you please show me where members of your household most often wash their hands?<br>*If there is not a designated place for handwashing, but instead a movable object like a basin, jerry can, or bucket select "Bucket, basin, or jerry can". If it is another object, please select "Other" and record response*	1 Observed (fixed place for washing - sink, tippy tap)<br>4 Observed, bucket, basin, or jerry can<br>2 Not observed, not in dwelling/plot/yard<br>3 Not observed, no permission to see<br>5 Other (specify)

Field	Question	Answer
WS11 *(required)*	If other, describe	
WS12 *(required)*	OBSERVATION.<br><br>Observe presence of water at the place for handwashing.<br><br>Verify by checking the tap/pump, or basin, bucket, water container or similar objects for presence of water.<br>*If there is a bucket, basin or other type of water container, examine to see whether water is present in the container. If you do not see water at the specific place for handwashing, code it as "Water not available"/"Ntamazi ahari"*	0 Yes, water is available<br>1 Water is not available
WS13a *(required)*	Do you have any soap or detergent or ash in your household for washing hands?	0 No<br>1 Yes<br>99 Don't Know
WS13b *(required)*	Is soap, detergent or ash present at the place for handwashing?<br><br>Record your observation.<br><br>Check all that apply.	0 Not present<br>1 Bar soap present<br>2 Detergent (powder/liquid/paste) present<br>3 Liquid soap present<br>4 Ash present
Social Support for Caregiver		
issb_note	The following questions are about help you get from different people in your life. Sometimes, people look to others for many kinds of support and assistance. When you need it, how often do you get any of the following forms of help or support from people in the month?	
issb1 *(required)*	How often can you find somebody to take care of people you live with or your property when you can't be there?	1 Never<br>2 A little<br>3 Sometimes<br>4 Quite a lot of the time<br>5 Nearly all the time<br>99 I don't know
issb2 *(required)*	How often is somebody there for you when you feel sad or lonely?	1 Never<br>2 A little<br>3 Sometimes<br>4 Quite a lot of the time<br>5 Nearly all the time<br>99 I don't know
issb3 *(required)*	How often is there someone to tell you what they did in a situation similar to what you're going through?	1 Never<br>2 A little<br>3 Sometimes<br>4 Quite a lot of the time<br>5 Nearly all the time<br>99 I don't know
issb4 *(required)*	How often is there someone to take you to do something fun to get your problems off your mind?	1 Never<br>2 A little<br>3 Sometimes<br>4 Quite a lot of the time<br>5 Nearly all the time<br>99 I don't know
issb5 *(required)*	How often is there someone in the community to help you find an opportunity to better your life?	1 Never<br>2 A little<br>3 Sometimes<br>4 Quite a lot of the time<br>5 Nearly all the time<br>99 I don't know
issb6 *(required)*	How often is there someone to appreciate or praise you for a job well done?	1 Never<br>2 A little<br>3 Sometimes<br>4 Quite a lot of the time<br>5 Nearly all the time<br>99 I don't know
issb7 *(required)*	How often is there someone to give you information on where you can go for help?	1 Never<br>2 A little<br>3 Sometimes<br>4 Quite a lot of the time<br>5 Nearly all the time<br>99 I don't know

Field	Question	Answer
issb8 *(required)*	How often is there someone to comfort you or tell you that problems will pass?	1 Never<br>2 A little<br>3 Sometimes<br>4 Quite a lot of the time<br>5 Nearly all the time<br>99 I don't know
issb9 *(required)*	How often is there someone to help you to find a direction for your life or a make a life decision regarding your future?	1 Never<br>2 A little<br>3 Sometimes<br>4 Quite a lot of the time<br>5 Nearly all the time<br>99 I don't know
issb10 *(required)*	How often is there someone to advise you about a situation you are dealing with?	1 Never<br>2 A little<br>3 Sometimes<br>4 Quite a lot of the time<br>5 Nearly all the time<br>99 I don't know
issb11 *(required)*	How often is there someone who can give/loan you one thousand Rwandan francs or more (1,000) when you need it?	1 Never<br>2 A little<br>3 Sometimes<br>4 Quite a lot of the time<br>5 Nearly all the time<br>99 I don't know
issb12 *(required)*	How often can you find someone to listen to you or talk about how you feel?	1 Never<br>2 A little<br>3 Sometimes<br>4 Quite a lot of the time<br>5 Nearly all the time<br>99 I don't know
issb13 *(required)*	How often is there someone who can lend you something (other than money) when you need it?	1 Never<br>2 A little<br>3 Sometimes<br>4 Quite a lot of the time<br>5 Nearly all the time<br>99 I don't know
issb14 *(required)*	How often is there someone to train you or teach you to do something when you need it?	1 Never<br>2 A little<br>3 Sometimes<br>4 Quite a lot of the time<br>5 Nearly all the time<br>99 I don't know
issb15 *(required)*	How often is there someone to make you laugh just to cheer you up?	1 Never<br>2 A little<br>3 Sometimes<br>4 Quite a lot of the time<br>5 Nearly all the time<br>99 I don't know
issb16 *(required)*	How often is there someone to join you for a prayer?	1 Never<br>2 A little<br>3 Sometimes<br>4 Quite a lot of the time<br>5 Nearly all the time<br>99 I don't know
issb17 *(required)*	How often is there someone to take care of you when you are sick or to take you to the hospital or to a traditional doctor if you need it?	1 Never<br>2 A little<br>3 Sometimes<br>4 Quite a lot of the time<br>5 Nearly all the time<br>99 I don't know
issb18 *(required)*	How often is there someone to come and help you with your chores: for example your housework or working your land or any other needed work?	1 Never<br>2 A little<br>3 Sometimes<br>4 Quite a lot of the time<br>5 Nearly all the time<br>99 I don't know

Field	Question	Answer
issb19 *(required)*	How often is there someone you can you call on to accompany you to visit your relatives who live far away?	1 Never<br>2 A little<br>3 Sometimes<br>4 Quite a lot of the time<br>5 Nearly all the time<br>99 I don't know
issb20 *(required)*	How often are you together with your friends?	1 Never<br>2 A little<br>3 Sometimes<br>4 Quite a lot of the time<br>5 Nearly all the time<br>99 I don't know
issb21 *(required)*	How often can you count on your family or friends to help you with your problems?	1 Never<br>2 A little<br>3 Sometimes<br>4 Quite a lot of the time<br>5 Nearly all the time<br>99 I don't know
issb22 *(required)*	How often do you feel comfortable enough to talk freely about your worries to others?	1 Never<br>2 A little<br>3 Sometimes<br>4 Quite a lot of the time<br>5 Nearly all the time<br>99 I don't know
issb23 *(required)*	How often do your family members and friends demand that you do something?	1 Never<br>2 A little<br>3 Sometimes<br>4 Quite a lot of the time<br>5 Nearly all the time<br>99 I don't know
issb24 *(required)*	How often is there someone to offer you a place to stay?	1 Never<br>2 A little<br>3 Sometimes<br>4 Quite a lot of the time<br>5 Nearly all the time<br>99 I don't know
issb25 *(required)*	How often is there someone to offer you advice and help you to understand or solve your problems?	1 Never<br>2 A little<br>3 Sometimes<br>4 Quite a lot of the time<br>5 Nearly all the time<br>99 I don't know
issb26 *(required)*	How often do people help you as much as they can?	1 Never<br>2 A little<br>3 Sometimes<br>4 Quite a lot of the time<br>5 Nearly all the time<br>99 I don't know
issb27 *(required)*	How often is there someone to give you something essential when you need it?	1 Never<br>2 A little<br>3 Sometimes<br>4 Quite a lot of the time<br>5 Nearly all the time<br>99 I don't know
issb28 *(required)*	How often is there someone who you can talk to when you need it?	1 Never<br>2 A little<br>3 Sometimes<br>4 Quite a lot of the time<br>5 Nearly all the time<br>99 I don't know
issb30 *(required)*	How often do people gather together to discuss problems they have in common?	1 Never<br>2 A little<br>3 Sometimes<br>4 Quite a lot of the time<br>5 Nearly all the time<br>99 I don't know

Field	Question	Answer
issb32 *(required)*	How often is there someone to help you get out of loneliness or hopelessness?	1 Never<br>2 A little<br>3 Sometimes<br>4 Quite a lot of the time<br>5 Nearly all the time<br>99 I don't know
issb33 *(required)*	How often is there someone to show you love?	1 Never<br>2 A little<br>3 Sometimes<br>4 Quite a lot of the time<br>5 Nearly all the time<br>99 I don't know
Medical Complications		
MC2 *(required)*	Does your child appear pale in the eyes, or weak, or past history of blood transfusions?	0 No<br>1 Yes<br>99 Don't Know
MC3 *(required)*	Does your child have trouble eating: such as problems chewing or swaollowing or choking or gagging while eating?	0 No<br>1 Yes<br>99 Don't Know
MC3_duration *(required)*	At what age did this problem start?<br>*Enter response in months. Enter "99" if they do not know.*	
MC4 *(required)*	Does your child have difficulty breathing on a daily basis, such as breathing fast, or chronic cough, or out of breath when walking?	0 No<br>1 Yes<br>99 Don't Know
MC5 *(required)*	Has your child ever had any siezures?	0 No<br>1 Yes<br>99 Don't Know
MC5_b *(required)*	Does your child currently experience siezures?	0 No<br>1 Yes<br>99 Don't Know
MC5_c *(required)*	Does your child currently take any medication for siezures, such as depakine or phenotoin?	0 No<br>1 Yes<br>99 Don't Know
readmit *(required)*	Was your child ever readmitted to the hospital after he/she was first discharged from the neonatal unit?	0 No<br>1 Yes<br>99 Don't Know
readmit_freq *(required)*	In total many times has your child been admitted to the hospital, not including his/her first time in neonatology?	
readmit_when *(required)*	When was your child first readmitted?	1 Within 1 month after discharge<br>2 Within 2-5 months after discharge<br>3 More than 6 months after discharge<br>99 Don't Know
Infant and young child feeding practices		
iycf1 *(required)*	Has {child_name} ever been breastfed?	0 No<br>1 Yes<br>99 Don't Know
iycf2 *(required)*	Is {child_name} still being breastfed?	0 No<br>1 Yes<br>99 Don't Know
diff_bf *(required)*	Did you ever experience difficulties breastfeeding {child_name}?	0 No<br>1 Yes<br>99 Don't Know
diff_bf_spec *(required)*	What types of difficulties did you experience while breastffeeding {child_name}?	1 Not enough breastmilk<br>2 Pain or other breast issues<br>3 Not enough time to breastfeed<br>4 He/she had difficulty attaching<br>5 He/she had difficulty swallowing<br>6 Other<br>99 Don't know
diff_bf_other *(required)*	Specify the other types of difficulties	
diff_bf_help_yn *(required)*	Did you receive any help for these breastfeeding difficulties?	0 No<br>1 Yes<br>99 Don't Know

Field	Question	Answer
diff_bf_help *(required)*	Where did you receive any help for these breastfeeding difficulties?	1 Friend/relative<br>2 Traditional practitioner<br>3 Ref. Hospital<br>4 Dist. Hospital<br>5 Health center<br>6 Health post<br>7 Community health worker<br>8 From an NGO (Save the Children, Caritas, Partners, or others)<br>9 Church<br>10 Other<br>11 Don't know
infant_formula *(required)*	Did you ever use infant formula to feed {child_name}?	0 No<br>1 Yes<br>99 Don't Know
formula_age *(required)*	At what age did you start giving infant formula?<br>*Enter age in months*	
formula_source *(required)*	Where did you get the infant formula?	1 Bought from a Private Business<br>2 From an NGO (Save the Children, Caritas, Partners, or others)<br>3 From a health facility<br>4 From the Government<br>6 Other<br>99 Don't Know
formula_source_other *(required)*	Describe other source of formula	
start_compfeed *(required)*	How old was {child_name} when you introduced other liquids or foods besides breastmilk or infant formula?	0 <1 months of age<br>1 1 month old<br>2 2 months old<br>3 3 months old<br>4 4 months old<br>5 5 months old<br>6 6 months old<br>7 7 months old<br>8 8 months old<br>9 9 months old<br>10 10 or more months old<br>99 Don't Know
iycf36 *(required)*	Why did you start to give {child_name} food and drink other than breast milk?<br>*Can select more than one response*	1 Advised by partner<br>2 Advised by my mother or mother-in-law<br>3 Advised by community health worker<br>4 The (index child) looked thin/under-nourished<br>5 The (index child) looked hungry and was crying.<br>6 Mother had to work and could not continue to breastfeed exclusively (work including farming, market, etc.)<br>7 Mother did not think she was producing enough breastmilk<br>8 Because child reached the age he/she is supposed to start eating food<br>9 Mother does not live at home<br>10 Other<br>99 I don't know
Infant and young child's feeding practices: checklist of liquids		
iycf6	Now I would like to ask you about (other) liquids that {child_name} may have had yesterday during the day or the night. I am interested to know whether {child_name} had the item even if combined with other foods.<br><br>Please include liquids consumed outside of your home.<br><br>Did {child_name} drink (name of item) yesterday during the day or the night:	

Field	Question	Answer
iycf7 *(required)*	Plain water?	0 No<br>1 Yes<br>99 Don't Know
iycf8 *(required)*	Juice or juice drinks?	0 No<br>1 Yes<br>99 Don't Know
icyf_soda *(required)*	Soda or fanta?	0 No<br>1 Yes<br>99 Don't Know
iycf10 *(required)*	Milk, such as tinned, powdered, or fresh animal milk?	0 No<br>1 Yes<br>99 Don't Know
iycf14 *(required)*	How many times did {child_name} drink milk?<br>*If 7 or more times, record "7". If unknown, record "99".*	
Dietary Diversity for Children 24+ mos		
dietdiv_instr	Now I would like to ask you about foods that {child_name} may have had yesterday during the day or the night. Again, I am interested to know whether {child_name} had the item even if combined with other foods.<br><br>Please include foods consumed outside of your home.<br><br>Did {child_name} eat (name of food) yesterday during the day or the night:	
dietdiv1 *(required)*	CEREALS: corn/maize, rice, wheat, sorghum or any other grains or foods made from these (e.g. bread, noodles, porridge, ugali, or other grain products)	0 No<br>1 Yes<br>99 Don't Know
dietdiv2 *(required)*	VITAMIN A RICH VEGETABLES AND TUBERS: pumpkin, carrots, squash, or sweet<br>potatoes that are orange inside + other<br>locally available vitamin A rich vegetables	0 No<br>1 Yes<br>99 Don't Know
dietdiv3 *(required)*	DARK GREEN LEAFY VEGETABLES: dodo, isombe, spinach, kale	0 No<br>1 Yes<br>99 Don't Know
dietdiv4 *(required)*	OTHER VEGETABLES: onions, tomatioes, cabbage, peppers, eggplant, beets, others	0 No<br>1 Yes<br>99 Don't Know
dietdiv5 *(required)*	VITAMIN A RICH FRUITS: ripe mangoes, ripe papaya, passion fruit, tree tomotatoes + other locally available vitamin A rich fruits	0 No<br>1 Yes<br>99 Don't Know
dietdiv6 *(required)*	OTHER FRUITS: other fruits, including wild fruits (watermelon, banana, pinepple, avocado, and guava)	0 No<br>1 Yes<br>99 Don't Know
dietdiv7 *(required)*	WHITE ROOTS AND TUBERS: white potatoes, white yams, white cassava, green banana/matooke, or other foods made from roots	0 No<br>1 Yes<br>99 Don't Know
dietdiv10 *(required)*	ORGAN MEAT: liver, kidney, heart or other organ meats or blood-based foods	0 No<br>1 Yes<br>99 Don't Know
dietdiv11 *(required)*	FLESH MEATS: beef, pork, lamb, goat, rabbit, wild game, chicken, duck, or other bird	0 No<br>1 Yes<br>99 Don't Know
dietdiv12 *(required)*	EGGS: chicken, duck, guinea fowl or any other<br>egg	0 No<br>1 Yes<br>99 Don't Know
dietdiv13 *(required)*	FISH: fresh or dried fish or shellfish (locally sambaza, sardines, small dried fish)	0 No<br>1 Yes<br>99 Don't Know
dietdiv14 *(required)*	LEGUMES, NUTS AND SEEDS: beans, peas, lentils, nuts, seeds or foods made from these	0 No<br>1 Yes<br>99 Don't Know
dietdiv15 *(required)*	MILK AND MILK PRODUCTS: milk, cheese, yogurt or other milk products	0 No<br>1 Yes<br>99 Don't Know
dietdiv16 *(required)*	OILS AND FATS: oil, fats or butter added to food or used for cooking	0 No<br>1 Yes<br>99 Don't Know
dietdiv17 *(required)*	SWEETS: sugar, honey, sweetened soda, sweetened<br>juice or sugary foods such as chocolates,<br>candies, cookies and cakes (include sugar added to tea)	0 No<br>1 Yes<br>99 Don't Know

Field	Question	Answer
dietdiv18 *(required)*	SPICES, CONDIMENTS, BEVERAGES: spices (black pepper, salt), condiments (soy sauce, hot sauce, pili pili, akabanga), coffee, tea, alcoholic beverages or local examples	0 No<br>1 Yes<br>99 Don't Know
dietdiv19 *(required)*	Did the child eat anything (meal or snack) OUTSIDE the home yesterday?	0 No<br>1 Yes<br>99 Don't Know
Meal Frequency for Child		
meal_freq_child *(required)*	How many times did {child_name} eat any solid, semi-solid, or soft foods yesterday during the day or night?	
meal_freq_adult *(required)*	Yesterday, how many times did the adults in this household eat?<br>*Enter "99" if the cargeiver does not know*	
Household food security		
d4	In the past 7 days, if there have been times when your household did not have enough food or money to buy food, how many days has your household had to…	
d5 *(required)*	Rely on less preferred and less expensive foods?<br>*Enter "99" if the cargeiver does not know*	
d6 *(required)*	Borrow food, or rely on help from a friend or relative?<br>*Enter "99" if the cargeiver does not know*	
d7 *(required)*	Limit portion size at mealtimes?<br>*Enter "99" if the cargeiver does not know*	
d8 *(required)*	Restrict consumption by adults in order for small children to eat?<br>*Enter "99" if the cargeiver does not know*	
d9 *(required)*	Reduce number of meals eaten in a day?<br>*Enter "99" if the cargeiver does not know*	
Months of Food Availability		
mahfp_screen *(required)*	In the last 12 months were there any specific months in which your household found it does not have enough food or money to buy food?	0 No<br>1 Yes<br>99 Don't Know
mahfp1 *(required)*	If yes, in which months did your household found it does not have enough food or money to buy food	22 June 2019<br>21 May 2019<br>20 April 2019<br>19 March 2019<br>18 February 2019<br>17 January 2019<br>16 December 2018<br>15 November 2018<br>1 October 2018<br>2 September 2018<br>3 August 2018<br>4 July 2018<br>5 June 2018<br>6 May 2018
mahfp2 *(required)*	Was this situation usual for your household at that time of year?	0 No<br>1 Yes<br>99 Don't Know
Caregiver Mental Health		
hscl_note	INSTRUCTIONS: The following are problems or signs of problems that people might have. I am going to read each one carefully and please tell us how many times it happened to you or stressed you last week including today.	
hscl1 *(required)*	Sudden fear, being frightened for no apparent reason	0 Not at all<br>1 A little<br>2 Quite a bit<br>3 Extremely<br>99 Don't Know
hscl2 *(required)*	Feeling scared/being afraid	0 Not at all<br>1 A little<br>2 Quite a bit<br>3 Extremely<br>99 Don't Know
hscl3 *(required)*	Fainting, feeling dizzy, feeling weak/lack of energy	0 Not at all<br>1 A little<br>2 Quite a bit<br>3 Extremely<br>99 Don't Know
hscl4 *(required)*	Anxious or feeling shaken	0 Not at all<br>1 A little<br>2 Quite a bit<br>3 Extremely<br>99 Don't Know

Field	Question	Answer
hscl5 *(required)*	Heart racing or jumping	0 Not at all<br>1 A little<br>2 Quite a bit<br>3 Extremely<br>99 Don't Know
hscl6 *(required)*	Shivering	0 Not at all<br>1 A little<br>2 Quite a bit<br>3 Extremely<br>99 Don't Know
hscl7 *(required)*	Feeling afraid or worried	0 Not at all<br>1 A little<br>2 Quite a bit<br>3 Extremely<br>99 Don't Know
hscl8 *(required)*	Headaches	0 Not at all<br>1 A little<br>2 Quite a bit<br>3 Extremely<br>99 Don't Know
hscl9 *(required)*	Spells of terror or panic	0 Not at all<br>1 A little<br>2 Quite a bit<br>3 Extremely<br>99 Don't Know
hscl10 *(required)*	Feeling restless, can't sit still	0 Not at all<br>1 A little<br>2 Quite a bit<br>3 Extremely<br>99 Don't Know
hscl11 *(required)*	Feeling tired/discouraged	0 Not at all<br>1 A little<br>2 Quite a bit<br>3 Extremely<br>99 Don't Know
hscl12 *(required)*	Blaming yourself for things	0 Not at all<br>1 A little<br>2 Quite a bit<br>3 Extremely<br>99 Don't Know
hscl13 *(required)*	Crying for nothing/ easily in tears	0 Not at all<br>1 A little<br>2 Quite a bit<br>3 Extremely<br>99 Don't Know
hscl14 *(required)*	Lack of interests in sex, no pleasure in sex	0 Not at all<br>1 A little<br>2 Quite a bit<br>3 Extremely<br>99 Don't Know
hscl15 *(required)*	Lack of appetite	0 Not at all<br>1 A little<br>2 Quite a bit<br>3 Extremely<br>99 Don't Know
hscl16 *(required)*	Difficulty in falling asleep or staying asleep	0 Not at all<br>1 A little<br>2 Quite a bit<br>3 Extremely<br>99 Don't Know
hscl17 *(required)*	Hopelessness/ no faith in the future	0 Not at all<br>1 A little<br>2 Quite a bit<br>3 Extremely<br>99 Don't Know

Field	Question	Answer	
hscl18 *(required)*	Feeling blue	0<br>1<br>2<br>3<br>99	Not at all<br>A little<br>Quite a bit<br>Extremely<br>Don't Know
hscl19 *(required)*	Isolation/loneliness	0<br>1<br>2<br>3<br>99	Not at all<br>A little<br>Quite a bit<br>Extremely<br>Don't Know
hscl20 *(required)*	Thoughts of ending your life<br>*[RISK OF HARM PROTOCOL QUESTION]. If participant responds quite a bit or extremely, express concern about this and follow up to assess severity.*	0<br>1<br>2<br>3<br>99	Not at all<br>A little<br>Quite a bit<br>Extremely<br>Don't Know
hscl21 *(required)*	Feeling trapped	0<br>1<br>2<br>3<br>99	Not at all<br>A little<br>Quite a bit<br>Extremely<br>Don't Know
hscl22 *(required)*	Feeling very worried	0<br>1<br>2<br>3<br>99	Not at all<br>A little<br>Quite a bit<br>Extremely<br>Don't Know
hscl23 *(required)*	Feeling like nothing interests you/ nothing is meaningful to you	0<br>1<br>2<br>3<br>99	Not at all<br>A little<br>Quite a bit<br>Extremely<br>Don't Know
hscl24 *(required)*	Everything requires lots of effort	0<br>1<br>2<br>3<br>99	Not at all<br>A little<br>Quite a bit<br>Extremely<br>Don't Know
hscl25 *(required)*	Feeling like you are not worthy/ you are worthless	0<br>1<br>2<br>3<br>99	Not at all<br>A little<br>Quite a bit<br>Extremely<br>Don't Know
Service Access			
ecd_attend *(required)*	Does {child_name} attend any organized learning or early childhood education programme, such as a private or government facility, including kindergarten or community child care?	0<br>1<br>99	No<br>Yes<br>Don't Know
ecd_hours *(required)*	In the past 7 days, about how many hours did {child_name} go to that place:		
growth_mon *(required)*	In the past month, has {child_name} seen a health provider or community health worker to measure his/her growth?	0<br>1<br>99	No<br>Yes<br>Don't Know
maln_prog *(required)*	Has your child ever been enrolled in a program due to malnutrition?<br>*This DOES NOT INCLUDE services at PDC. We are referring to malnutrition programs at hospital or health centers.*	0<br>1<br>99	No<br>Yes<br>Don't Know
maln_prog_type *(required)*	What type of program for malnutrition was your child enrolled in?	1<br>2<br>3<br>4<br>5<br>10	Health center program for severe malnutrition (OTP)<br>Health center program for moderate malnutrition (SFP)<br>Health center program (type not specified)<br>Hospitalized for malnutrition program<br>Community education programs (growth monitoring in community, cooking demonstrations)<br>Other
maln_prog_current *(required)*	Is your child currently enrolled in this program?	0<br>1<br>99	No<br>Yes<br>Don't Know

Field	Question	Answer
		99 Don't Know
maln_prog_time *(required)*	When was your child last enrolled in the program?	1 <6 months ago<br>2 6 to 11 months ago<br>3 > 12 months (1 year) ago<br>10 Other
maln_prog_time_other *(required)*	Enter when the child was last enrolled<br>*Ideally month and year. Enter only year if caregiver is not sure of the month. Enter "99" if caregiver does not know.*	
Child Discipline		
cd_note	INSTRUCTIONS: Adults use certain ways to teach children the right behaviour or to address a behaviour problem. I will read various methods that are used and I want you to tell me if you or anyone else in your household has used this method in the past 30 days with the index child:	
cd1 *(required)*	Deprived the child of things that the child desired because of misbehavior	0 No<br>1 Yes<br>99 Don't Know
cd3 *(required)*	Explained why something (the behavior) was wrong	0 No<br>1 Yes<br>99 Don't Know
cd4 *(required)*	Shook the child when angry with him/her	0 No<br>1 Yes<br>99 Don't Know
cd5 *(required)*	Shouted, yelled, or screamed at the child	0 No<br>1 Yes<br>99 Don't Know
cd6 *(required)*	Gave the child work as a form of punishment, so that he/she stopped misbehaving.	0 No<br>1 Yes<br>99 Don't Know
cd7 *(required)*	Spanked, hit, or slapped him/her on the bottom with bare hand	0 No<br>1 Yes<br>99 Don't Know
cd8 *(required)*	Hit him/her on the bottom or elsewhere on the body with something like a belt, whip, stick, or other hard object	0 No<br>1 Yes<br>99 Don't Know
cd9 *(required)*	Called him/her dumb, lazy, or another name like that	0 No<br>1 Yes<br>99 Don't Know
cd10 *(required)*	Hit or slapped him/her on the face, head, or ears	0 No<br>1 Yes<br>99 Don't Know
cd11 *(required)*	Hit or slapped him/her on the hand, arm, or leg	0 No<br>1 Yes<br>99 Don't Know
cd12 *(required)*	Beat him/her up, that is hit him/her over and over as hard as one could<br>*[RISK OF HARM PROTOCOL QUESTION]. This item indicates potential concern about abuse. If the caregiver says YES, express concern about this and follow up to assess severity.*	0 No<br>1 Yes<br>99 Don't Know
cd13 *(required)*	Do you believe that in order to bring up, raise, or educate a child properly, the child needs to be physically punished?	0 No<br>1 Yes<br>99 Don't Know
ECD Index - MICS		
picture_books *(required)*	How many children's books or picture books do you have for {child_name}?<br>*Enter "99" if caregiver does not know.*	
ecd_index_note	I am interested in learning about the things that {child_name} plays with when he/she is at home.<br>*Note to interviewer: If the respondent says "YES" to the categories below then probe to learn specifically what the child plays with to ascertain the response.*	
homemade_toys *(required)*	Does he/she play with: homemade toys (such as dolls, cars, or other toys made at home)?	0 No<br>1 Yes<br>99 Don't Know
purchased_toys *(required)*	Does he/she play with: toys from a shop or manufactured toys?	0 No<br>1 Yes<br>99 Don't Know
household_toys *(required)*	Does he/she play with: household objects (such as bowls or pots) or objects found outside (such as sticks, rocks, animal shells or leaves)?	0 No<br>1 Yes<br>99 Don't Know
inad_alone *(required)*	On how many days in the past week was {child_name} left alone for more than an hour?<br>*If none enter 0.*	
inad_child *(required)*	On how many days in the past week was {child_name} left in the care of another child, that is, someone less than 10 years old, for more than an hour?<br>*If none enter 0.*	
ECD Index - MICS		

Field	Question	Answer
ecd_index_note2	In the past 3 days, did you or any household member age 15 or over engage in any of the following activities with {child_name}:<br><br>*If yes, ask:<br>*Who engaged in this activity with {child_name}?<br><br>*RECORD ALL MENTIONED.<br>**DO NOT READ RESPONSE OPTIONS OUT LOUD.*	
ecd_index1 *(required)*	Read books to or looked at picture books with {child_name}?<br>*Select all that are mentioned.*	1 Mother<br>2 Father<br>3 Other<br>4 No one
ecd_index2 *(required)*	Told stories to {child_name}?<br>*Select all that are mentioned.*	1 Mother<br>2 Father<br>3 Other<br>4 No one
ecd_index3 *(required)*	Sang songs to {child_name} or with {child_name}, including lullabies?<br>*Select all that are mentioned.*	1 Mother<br>2 Father<br>3 Other<br>4 No one
ecd_index4 *(required)*	Took {child_name} outside the home, compound, yard or enclosure?<br>*Select all that are mentioned.*	1 Mother<br>2 Father<br>3 Other<br>4 No one
ecd_index5 *(required)*	Played with {child_name}?<br>*Select all that are mentioned.*	1 Mother<br>2 Father<br>3 Other<br>4 No one
ecd_index6 *(required)*	Named, counted, or drew things to or with {child_name}?<br>*Select all that are mentioned.*	1 Mother<br>2 Father<br>3 Other<br>4 No one
LONG FORM: MOTOR, COGNITIVE, LANGUAGE, & SOCIAL-EMOTIONAL		
LF_note	**Now I am going to ask you about the types of things your child is currently able to do. Please answer "yes" or "no" to these questions. If you are unsure, you can also answer by saying "don't know." Please keep in mind that children learn and grow at different rates, so it is fine if your child can't yet do these things. Some of these skills children only achieve at older ages. If there is any question you feel uncomfortable answering, please let me know and we can move to the next question.**	
LF1 *(required)*	When lying on his/her back, does the child move his/her arms and legs?<br>*Start point for 0-5 months*	1 Yes<br>0 No<br>0.1 Don't Know/No response
LF2 *(required)*	Does the child bring his/her hand to his/her mouth?	1 Yes<br>0 No<br>0.1 Don't Know/No response
LF3 *(required)*	Does the child laugh?	1 Yes<br>0 No<br>0.1 Don't Know/No response
LF4 *(required)*	Does the child smile when others smile at him/her?	1 Yes<br>0 No<br>0.1 Don't Know/No response
LF5 *(required)*	Does the child sometimes suck his/her thumb or fingers?	1 Yes<br>0 No<br>0.1 Don't Know/No response
LF6 *(required)*	Does the child grasp onto a small object (e.g., your finger, a spoon) when put in his/her hand?<br>*Start point for 6-11 months*	1 Yes<br>0 No<br>0.1 Don't Know/No response
LF7 *(required)*	Can the child bring his/her hands together?	1 Yes<br>0 No<br>0.1 Don't Know/No response
LF8 *(required)*	Does the child recognize you or other family members (e.g., smile when they enter a room or move toward them)?	1 Yes<br>0 No<br>0.1 Don't Know/No response
LF9 *(required)*	Does the child hold his/her hands in fists all the time?	0 Yes<br>1 No<br>0.1 Don't Know/No response

Field	Question	Answer
LF10 *(required)*	Does the child show interest in new objects that are put in front of him/her by reaching out for them?	1 Yes<br>0 No<br>0.1 Don't Know/No response
LF11 *(required)*	Can the child roll from his/her back to stomach, or stomach to back, on his/her own?<br>*Start point for 12-17 months*	1 Yes<br>0 No<br>0.1 Don't Know/No response
LF12 *(required)*	Does the child show interest in new objects by trying to put them in his/her mouth?	1 Yes<br>0 No<br>0.1 Don't Know/No response
LF13 *(required)*	Does the child often show affection toward others (e.g., hugging parents, brothers, or sisters)?	1 Yes<br>0 No<br>0.1 Don't Know/No response
LF14 *(required)*	Can the child pick up a small object (e.g., a small toy or small stone) using just one hand?	1 Yes<br>0 No<br>0.1 Don't Know/No response
LF15 *(required)*	Does the child look for an object of interest when it is removed from sight or hidden from him/her (e.g., put under a cover, behind another object)?	1 Yes<br>0 No<br>0.1 Don't Know/No response
LF16 *(required)*	When lying on his/her back, does the child grab his/her feet?	1 Yes<br>0 No<br>0.1 Don't Know/No response
LF17 *(required)*	Can the child make simple sounds like "ba," "da," or "do?"	1 Yes<br>0 No<br>0.1 Don't Know/No response
LF18 *(required)*	When lying on his/her stomach, can the child hold his/her head and chest off the ground using only his/her hands and arms for support?	1 Yes<br>0 No<br>0.1 Don't Know/No response
LF19 *(required)*	Does the child play by tapping an object on the ground or a table?	1 Yes<br>0 No<br>0.1 Don't Know/No response
LF20 *(required)*	Can the child hold him/herself in a sitting position without help or support for longer than a few seconds?	1 Yes<br>0 No<br>0.1 Don't Know/No response
LF21 *(required)*	Does the child intentionally move or change his/her position to get objects that are out of reach?	1 Yes<br>0 No<br>0.1 Don't Know/No response
LF22 *(required)*	Does the child look at an object when someone says "look!" and points to it?	1 Yes<br>0 No<br>0.1 Don't Know/No response
LF23 *(required)*	Does the child recognize his/her name or nickname? That is, does he/she respond differently to his/her name than to other sounds or words?	1 Yes<br>0 No<br>0.1 Don't Know/No response
LF24 *(required)*	When you talk to the child, does he/she respond by making a sound (e.g., "ba," "da," or "do") or by saying a word?	1 Yes<br>0 No<br>0.1 Don't Know/No response
LF25 *(required)*	Can the child crawl, roll, or scoot forward on his/her own?	1 Yes<br>0 No<br>0.1 Don't Know/No response
LF26 *(required)*	Can the child pick up and eat small pieces of food with his/her fingers?	1 Yes<br>0 No<br>0.1 Don't Know/No response
LF27 *(required)*	Can the child transfer a small object (e.g., a small toy or small stone) from one hand to the other?	1 Yes<br>0 No<br>0.1 Don't Know/No response
LF28 *(required)*	Does the child clap his/her hands together?	1 Yes<br>0 No<br>0.1 Don't Know/No response
LF29 *(required)*	Can the child maintain a standing position while holding on to a person or object (e.g., wall or furniture)?<br>*Start point for 18-23 months*	1 Yes<br>0 No<br>0.1 Don't Know/No response
LF30 *(required)*	Can the child use gestures to indicate what he/she wants (e.g., put arms up to indicate that he/she wants to be held, or point to water)?	1 Yes<br>0 No<br>0.1 Don't Know/No response

Field	Question	Answer
LF31 *(required)*	Can the child pick up a small object (e.g., a small toy or small stone) with just his/her thumb and a finger?	1 Yes<br>0 No<br>0.1 Don't Know/No response
LF32 *(required)*	Can the child pick up and drop a small object (e.g., a small toy or small stone) into a bucket or bowl while sitting?	1 Yes<br>0 No<br>0.1 Don't Know/No response
LF33 *(required)*	Can the child throw a small ball or small stone in a forward direction using his/her hand?	1 Yes<br>0 No<br>0.1 Don't Know/No response
LF34 *(required)*	Can the child walk several steps while holding on to a person or object (e.g., wall or furniture)?	1 Yes<br>0 No<br>0.1 Don't Know/No response
LF35 *(required)*	Can the child say one or more words (e.g., names like "Mama" or "ba" for "ball")?	1 Yes<br>0 No<br>0.1 Don't Know/No response
LF36 *(required)*	Can the child maintain a standing position on his/her own, without holding on or receiving support?	1 Yes<br>0 No<br>0.1 Don't Know/No response
LF37 *(required)*	Can the child follow simple directions (e.g., “Stand up" or "Come here”)?	1 Yes<br>0 No<br>0.1 Don't Know/No response
LF38 *(required)*	Does the child watch what other children do and try to copy them?	1 Yes<br>0 No<br>0.1 Don't Know/No response
LF39 *(required)*	Can the child sit or play on his/her own for at least 20 minutes?<br>*Start point for 24-29 months*	1 Yes<br>0 No<br>0.1 Don't Know/No response
LF40 *(required)*	Can the child walk several steps on his/her own, without holding on or receiving support?	1 Yes<br>0 No<br>0.1 Don't Know/No response
LF41 *(required)*	Can the child bend down to the ground and stand up again without falling and without holding onto a person or object?	1 Yes<br>0 No<br>0.1 Don't Know/No response
LF42 *(required)*	Does the child ask you for help using signs or words when he/she cannot do something on his/her own (e.g., to reach an object up high)?	1 Yes<br>0 No<br>0.1 Don't Know/No response
LF43 *(required)*	Does the child try to repeat sounds or words said by other people?	1 Yes<br>0 No<br>0.1 Don't Know/No response
LF44 *(required)*	Can the child climb onto an object such as a chair or bench?	1 Yes<br>0 No<br>0.1 Don't Know/No response
LF45 *(required)*	Can the child figure out how to turn a spoon or object if you give it to him/her the wrong way around?	1 Yes<br>0 No<br>0.1 Don't Know/No response
LF46 *(required)*	Does the child stop at least briefly when told “no” or “stop that"?	1 Yes<br>0 No<br>0.1 Don't Know/No response
LF47 *(required)*	Can the child kick a ball or other round object forward using his/her foot?	1 Yes<br>0 No<br>0.1 Don't Know/No response
LF48 *(required)*	Can the child point to a person or object when asked (e.g., “Where is mama?" or "Where is the ball?")?	1 Yes<br>0 No<br>0.1 Don't Know/No response
LF49 *(required)*	Can the child drink from a cup (without a lid) on his/her own without spilling?	1 Yes<br>0 No<br>0.1 Don't Know/No response
LF50 *(required)*	Does the child imitate animal or other sounds (e.g., "vroom" for a car, "moo" for a cow)?	1 Yes<br>0 No<br>0.1 Don't Know/No response
LF51 *(required)*	Can the child run more than a few steps without falling or bumping into objects?	1 Yes<br>0 No<br>0.1 Don't Know/No response

Field	Question	Answer
LF52 *(required)*	Can the child draw a line or shape on paper with a pen or crayon, or in the dirt with a stick?<br>*Start point for 30-35 months*	1 Yes<br>0 No<br>0.1 Don't Know/No response
LF53 *(required)*	Can the child answer simple questions (e.g., "Do you want water?") by saying "yes" or "no", rather than nodding?	1 Yes<br>0 No<br>0.1 Don't Know/No response
LF54 *(required)*	Can the child stack three or more small objects (e.g., blocks, cups, bottle caps) on top of each other?	1 Yes<br>0 No<br>0.1 Don't Know/No response
LF55 *(required)*	Does the child imitate others' behaviors (e.g., washing hands or dishes)?	1 Yes<br>0 No<br>0.1 Don't Know/No response
LF56 *(required)*	Does the child sometimes share things (e.g., food, toys) with others without being told?	1 Yes<br>0 No<br>0.1 Don't Know/No response
LF57 *(required)*	Can the child follow orders or instructions that have more than one part (e.g., "Go get water and go to bed")?	1 Yes<br>0 No<br>0.1 Don't Know/No response
LF58 *(required)*	Can the child say five or more separate words (e.g., names like "Mama" or objects like "ball")?	1 Yes<br>0 No<br>0.1 Don't Know/No response
LF59 *(required)*	Is the child kind to younger children (e.g., speaks to them nicely and touches them gently)?	1 Yes<br>0 No<br>0.1 Don't Know/No response
LF60 *(required)*	Can the child walk on an uneven surface (e.g., a bumpy or steep road) without falling?	1 Yes<br>0 No<br>0.1 Don't Know/No response
LF61 *(required)*	Does the child listen to someone telling a story with interest?	1 Yes<br>0 No<br>0.1 Don't Know/No response
LF62 *(required)*	Can the child ask for something (e.g., food, water) by name when he/she wants it?	1 Yes<br>0 No<br>0.1 Don't Know/No response
LF63 *(required)*	Does the child involve others in play (i.e., play interactive games with other children)?	1 Yes<br>0 No<br>0.1 Don't Know/No response
LF64 *(required)*	Can the child correctly name at least one family member other than mom and dad (e.g., name of brother, sister, aunt, uncle)?	1 Yes<br>0 No<br>0.1 Don't Know/No response
LF65 *(required)*	Does the child play by pretending objects are something else (e.g., imagining a bottle is a doll, a stone is a car, or a spoon is an airplane)?	1 Yes<br>0 No<br>0.1 Don't Know/No response
LF66 *(required)*	Does the child show sympathy or look concerned when others are hurt or sad?	1 Yes<br>0 No<br>0.1 Don't Know/No response
LF67 *(required)*	Can the child walk backwards?	1 Yes<br>0 No<br>0.1 Don't Know/No response
LF68 *(required)*	Does the child show curiosity to learn new things (e.g., by asking questions or exploring a new area)?	1 Yes<br>0 No<br>0.1 Don't Know/No response
LF69 *(required)*	Can the child feed him/herself using a spoon or other utensil without spilling?	1 Yes<br>0 No<br>0.1 Don't Know/No response
LF70 *(required)*	Can the child concentrate on one task (e.g., playing with friends, eating meal) for 20 minutes?	1 Yes<br>0 No<br>0.1 Don't Know/No response
LF71 *(required)*	Does the child know the names of at least two body parts (e.g., arm, eye, or nose)?	1 Yes<br>0 No<br>0.1 Don't Know/No response
LF72 *(required)*	If you show the child an object he/she knows well (e.g., a cup or animal), can he/she consistently name it?	1 Yes<br>0 No<br>0.1 Don't Know/No response

Field	Question	Answer
LF73 *(required)*	Can the child speak using short sentences of two words that go together (e.g., “Mama go” or "Dada eat")?	1 Yes<br>0 No<br>0.1 Don't Know/No response
LF74 *(required)*	Can the child use a tool (e.g., a stick or spoon) to reach objects that are far away?	1 Yes<br>0 No<br>0.1 Don't Know/No response
LF75 *(required)*	Can the child indicate when he/she needs to go to the toilet?	1 Yes<br>0 No<br>0.1 Don't Know/No response
LF76 *(required)*	Can the child say ten or more separate words (e.g., names like "Mama" or objects like "ball")?	1 Yes<br>0 No<br>0.1 Don't Know/No response
LF77 *(required)*	Can the child remove an item of clothing (e.g., take off his/her shirt)?	1 Yes<br>0 No<br>0.1 Don't Know/No response
LF78 *(required)*	Can the child tell you when he/she is tired or hungry?	1 Yes<br>0 No<br>0.1 Don't Know/No response
LF79 *(required)*	Does the child usually finish an activity he/she enjoys (e.g., a game or book)?	1 Yes<br>0 No<br>0.1 Don't Know/No response
LF80 *(required)*	Can the child easily switch back and forth between activities (e.g., go back to a game after being interrupted)?	1 Yes<br>0 No<br>0.1 Don't Know/No response
LF81 *(required)*	Can the child sing a short song or repeat parts of a rhyme from memory by him/herself?	1 Yes<br>0 No<br>0.1 Don't Know/No response
LF82 *(required)*	Can the child jump with both feet leaving the ground?	1 Yes<br>0 No<br>0.1 Don't Know/No response
LF83 *(required)*	Can the child speak using sentences of three or more words that go together (e.g., "I want water" or "The house is big")?	1 Yes<br>0 No<br>0.1 Don't Know/No response
LF84 *(required)*	Can the child whisper?	1 Yes<br>0 No<br>0.1 Don't Know/No response
LF85 *(required)*	Does the child greet neighbors or other people he/she knows without being told (e.g., by saying hello or gesturing hello)?	1 Yes<br>0 No<br>0.1 Don't Know/No response
LF86 *(required)*	Can the child unscrew the lid from a bottle or jar?	1 Yes<br>0 No<br>0.1 Don't Know/No response
LF87 *(required)*	Can the child correctly ask questions using any of the words "what," "which," "where," or "who"?	1 Yes<br>0 No<br>0.1 Don't Know/No response
LF88 *(required)*	Can the child correctly use any of the words "I," "you," "she," or "he" (e.g., "I go to store," or "He eats rice")?	1 Yes<br>0 No<br>0.1 Don't Know/No response
LF89 *(required)*	Does the child pronounce most of his/her words correctly?	1 Yes<br>0 No<br>0.1 Don't Know/No response
LF90 *(required)*	Can the child count up to five objects (e.g., fingers, people)?	1 Yes<br>0 No<br>0.1 Don't Know/No response
LF91 *(required)*	Does the child ask about familiar people other than parents when they are not there (e.g., "Where is the neighbor?")?	1 Yes<br>0 No<br>0.1 Don't Know/No response
LF92 *(required)*	If you show the child two objects or people of different size, can he/she tell you which one is the big one and which is the small one?	1 Yes<br>0 No<br>0.1 Don't Know/No response
LF93 *(required)*	Can the child stand on one foot for several seconds without holding on to a person or object (e.g., wall or furniture)?	1 Yes<br>0 No<br>0.1 Don't Know/No response

Field	Question	Answer
LF94 *(required)*	Can the child identify at least one color (e.g., red, blue, yellow)?	1 Yes<br>0 No<br>0.1 Don't Know/No response
LF95 *(required)*	Does the child regularly use describing words such as "fast," "short," "hot," "fat," or "beautiful" correctly?	1 Yes<br>0 No<br>0.1 Don't Know/No response
LF96 *(required)*	If you point to an object, can the child correctly use the words "on," "in," or "under" to describe where it is (e.g., "The cup is on the table" instead of "The cup is in the table.")	1 Yes<br>0 No<br>0.1 Don't Know/No response
LF97 *(required)*	Can the child explain in words what common objects like a cup or chair are used for?	1 Yes<br>0 No<br>0.1 Don't Know/No response
LF98 *(required)*	Can the child dress him/herself (e.g., put on his/her pants and shirt without help)?	1 Yes<br>0 No<br>0.1 Don't Know/No response
LF99 *(required)*	Does the child ask "why" questions (e.g., "Why are you tall?")?	1 Yes<br>0 No<br>0.1 Don't Know/No response
LF100 *(required)*	If you ask the child to give you three objects (e.g., stones, beans), does the child give you the correct amount?	1 Yes<br>0 No<br>0.1 Don't Know/No response
LF101 *(required)*	Does the child usually put objects or toys back where they belong after using them?	1 Yes<br>0 No<br>0.1 Don't Know/No response
LF102 *(required)*	Does the child frequently act impulsively or without thinking (e.g., running into the street without looking)?	0 Yes<br>1 No<br>0.1 Don't Know/No response
LF103 *(required)*	Does the child sometimes save things like candy or new toys for the future?	1 Yes<br>0 No<br>0.1 Don't Know/No response
LF104 *(required)*	Can the child say what others like or dislike (e.g., "Mama doesn't like fruit," "Papa likes football")?	1 Yes<br>0 No<br>0.1 Don't Know/No response
LF105 *(required)*	Can the child fasten and unfasten buttons without help?	1 Yes<br>0 No<br>0.1 Don't Know/No response
LF106 *(required)*	Can the child talk about things that will happen in the future using correct language (e.g., "Tomorrow he will attend school" or "Next week we will go to the market")?	1 Yes<br>0 No<br>0.1 Don't Know/No response
LF107 *(required)*	Can the child talk about things that have happened in the past using correct language (e.g., "Yesterday I played with my friend" or "Last week she went to the market")?	1 Yes<br>0 No<br>0.1 Don't Know/No response
LF108 *(required)*	Does the child know the names of any letters (e.g., A, B, C)?	1 Yes<br>0 No<br>0.1 Don't Know/No response
LONG FORM: MENTAL HEALTH		
LMH_note	** We have just a few more questions to answer. The rules for these are the same. Please continue to answer with "yes", "no," or "don't know."**<br><br>*Always start with the first question. Ask caregivers to answer all questions in this section.*	
LMH1 *(required)*	Does the child often cry for no reason (e.g., when he/she is not hungry or tired)?	0 Yes<br>1 No<br>0.1 Don't Know/No response
LMH2 *(required)*	Does the child frequently complain of headaches or stomachaches when he/she is not physically ill?	0 Yes<br>1 No<br>0.1 Don't Know/No response
LMH3 *(required)*	Does the child become upset when you are not with him/her?	0 Yes<br>1 No<br>0.1 Don't Know/No response
LMH4 *(required)*	Does the child become very shy, afraid, or upset around strangers, even when you're with him/her?	0 Yes<br>1 No<br>0.1 Don't Know/No response
LMH5 *(required)*	Does the child cling excessively to his/her caregiver, even in a safe setting?	0 Yes<br>1 No<br>0.1 Don't Know/No response

Field	Question	Answer
LMH6 *(required)*	Can the child sit still when asked to by an adult (e.g., for two minutes)?	1 Yes<br>0 No<br>0.1 Don't Know/No response
LMH7 *(required)*	Does the child cry or whine when he/she is made to wait for something he/she wants (e.g., toy or food)?	0 Yes<br>1 No<br>0.1 Don't Know/No response
LMH8 *(required)*	Does the child often kick, bite, or hit other children or adults?	0 Yes<br>1 No<br>0.1 Don't Know/No response
LMH9 *(required)*	Does the child become extremely withdrawn or shy in new situations?	0 Yes<br>1 No<br>0.1 Don't Know/No response
CHILD DISABILITY		
da_intro	Now I would like to talk to you more about ({child_name})'s health condition. This will take only a few minutes.	
disability1 *(required)*	Compared with other children, does or did ({child_name}) have any serious delay in sitting standing, or walking?	0 No<br>1 Yes<br>99 Don't Know
disability2 *(required)*	Compared with other children, does ({child_name}) have difficulty seeing, either in the daytime or at night?	0 No<br>1 Yes<br>99 Don't Know
disability3 *(required)*	Does ({child_name}) appear to have any difficulty hearing (uses hearing aid, hears with difficulty or completely deaf)?	0 No<br>1 Yes<br>99 Don't Know
disability4 *(required)*	When you tell ({child_name}) to do something, does he/she seem to understand what you are saying?	0 No<br>1 Yes<br>99 Don't Know
disability5 *(required)*	Does ({child_name}) have difficulty in walking or moving his/her arms or does he/she have weakness and/or stiffness in the arms or legs?	0 No<br>1 Yes<br>99 Don't Know
disability6 *(required)*	Does ({child_name}) sometimes have fits, become rigid, or lose consciousness?	0 No<br>1 Yes<br>99 Don't Know
disability7 *(required)*	Does ({child_name}) learn to do things like other children his/her age?	0 No<br>1 Yes<br>99 Don't Know
disability8 *(required)*	Does ({child_name}) speak at all (can he/she make him or herself understood in words; can he/she say any recognizable words)?	0 No<br>1 Yes<br>99 Don't Know
disability9 *(required)*	Is ({child_name})'s speech in any way different from normal (not clear enough to be understood by people other than the immediate family)?	0 No<br>1 Yes<br>99 Don't Know
disability10 *(required)*	Can ({child_name}) name at least one object (for example, an animal, a toy, a cup, a spoon)?	0 No<br>1 Yes<br>99 Don't Know
disability11 *(required)*	Compared with other children of the same age, does ({child_name}) appear in any way mentally backward, dull or slow?	0 No<br>1 Yes<br>99 Don't Know
Ages and Stages Questionnaire		
ASQ_note	The ASQ is a copyrighted measure available from https://agesandstages.com/	
Check Out		
F_ID_check *(required)*	Enter family's Study ID Code	
comments	Please enter any comments about the interview that you would like to share	

Study Questionnaire
in Kinyarwanda

Kirehe-PDC-FU

Field	Question	Answer
Child's Information		
child_sex	Andika igitsina cy'umwana<br>*Enter only if the sex of the child from EMR needs correction.*	0 Abagabo<br>1 Abagore
mult_birth *(required)*	Ese umwana avuka yari umwe cyangwa ari benshi (urugero, impanga cyangwa ari batatu)?	1 Yavutse ari umwe<br>2 Bavutse ari impanga<br>3 Bavutse ari batatu<br>99 Simbizi
bday *(required)*	Umwana {child_name} yavutse ryari?<br>*Verify the date from the birth registration if it was available. Or mutuelle/immunization card if available.*	
Caregiver Information		
cg_sex *(required)*	Andika igitsina cy'umubyeyi	0 Abagabo<br>1 Abagore
rel *(required)*	Ni irihe sano mufitanye n'umwana?	1 Nyina umubyara<br>2 Ise umubyara<br>3 Umubyeyi w'umugore umurera utaramubyaye<br>4 Umubyeyi w'umugabo umurera utaramubyaye<br>5 Muka Se<br>6 Umugabo wa nyina<br>7 Nyirasenge, Nyina wabo/Nyirarume, Se wabo<br>8 Sekuru/Nyirakuru<br>9 Irindi
rel_other *(required)*	Niba irindi, sobanura:	
age *(required)*	Wari wujuje imyaka ingahe ku itariki y'amavuko yawe iheruka?	
Information about the Caregiver		
Ed1 *(required)*	Wigeze ujya mu ishuli?	0 Oya<br>1 Yego<br>99 Simbizi
Ed2 *(required)*	Ni uwuhe mwaka w'amashuri warangije?	0 Nta na rimwe<br>1 P1<br>2 P2<br>3 P3<br>4 P4<br>5 P5<br>6 P6<br>16 P7<br>17 P8<br>18 P9<br>7 S1<br>8 S2<br>9 S3<br>10 S4<br>11 S5<br>12 S6<br>13 Amashuli makuru na kaminuza<br>14 Impamyabumenyi ya kaminuza<br>15 I seminari/Ishuli ry'abihayimana gusa<br>20 Ikindi
Ed2_other *(required)*	Niba ibindi, sobanura:	
Ed5 *(required)*	Waba ushobora gusoma no kwandika?	0 Oya<br>1 Yego<br>99 Simbizi
Mar1 *(required)*	Kuri ubu mwaba mwarashyingiranywe byemewe n'amategeko cyangwa mubana gusa nk'abantu bashakanye?	1 Yego, nashyingirwe byemewe n'amategeko<br>2 yego tubana muburyo butemewe n'amategeko<br>0 Oya ntitubana<br>99 Simbizi
Mar2 *(required)*	Uwo mwashakanye murabana ubu cyangwa hari ahandi aba?	1 Yego, barabana<br>0 Oya, aba ahandi<br>99 Simbizi

Field	Question	Answer
Family Composition		
hh_size *(required)*	Bose hamwe ni abantu bangahe baba mu rugo iwanyu?	
hh_u17 *(required)*	Muri uru rugo hari abana bangahe bari munsi y'imyaka 17?	
hh_u5 *(required)*	Udakuyemo umwana twaganiriyeho ({child_name}), muri uru rugo hari abana bangahe bari munsi y'imyaka 5?	
father_edu *(required)*	Se w'umwana {child_name}) yagarukiye mu wa kangahe?	0 Nta na rimwe<br>1 P1<br>2 P2<br>3 P3<br>4 P4<br>5 P5<br>6 P6<br>16 P7<br>17 P8<br>18 P9<br>7 S1<br>8 S2<br>9 S3<br>10 S4<br>11 S5<br>12 S6<br>13 Amashuli makuru na kaminuza<br>14 Impamyabumenyi ya kaminuza<br>15 I seminari/Ishuli ry'abihayimana gusa<br>20 Ikindi
father_home *(required)*	Ese mubana na se w'umwana wamubyaye?	0 Oya<br>1 Yego<br>99 Simbizi
father_age *(required)*	Se w'umwana ({child_name}) yari yujuje imyaka ingahe ku isabukuru ye y'amavuko iheruka?<br>*Enter 999 if don't know*	
home4 *(required)*	Se w'umwana ajya afata umwanya wo kwita ku mwana buri munsi? Urugero nko kuganira, gutemberana, cg gukina n'umwana?<br>*In a home setting in which the mother is the primary caregiver, interactions with the father, or father figure such as a grandfather, uncle, step-father, etc. living in the home, allow the child to experience variety in social, emotional, and play interactions. Score "Yes" if the father regularly spends time with the child. Score "No" if this never or rarely happens, even if this is because the father is often away for work or other reasons.*	0 Oya<br>1 Yego<br>99 Simbizi
mother_edu *(required)*	Ni ikihe cy'icyiciro cyo hejuru cy'amashuri nyina wa ({child_name}) yarangije?	0 Nta na rimwe<br>1 P1<br>2 P2<br>3 P3<br>4 P4<br>5 P5<br>6 P6<br>16 P7<br>17 P8<br>18 P9<br>7 S1<br>8 S2<br>9 S3<br>10 S4<br>11 S5<br>12 S6<br>13 Amashuli makuru na kaminuza<br>14 Impamyabumenyi ya kaminuza<br>15 I seminari/Ishuli ry'abihayimana gusa<br>20 Ikindi
mother_home *(required)*	Ese ubana na nyina wa ({child_name})?	0 Oya<br>1 Yego<br>99 Simbizi
mother_age *(required)*	Nyina wa ({child_name}) yari yujuje imyaka ingahe ku isabukuru ye y'amavuko iheruka?<br>*Enter 999 if don't know*	
PDC Feedback		

Field	Question	Answer
pdc_current *(required)*	Ese uracyajyana umwana wawe muri PDC* ukurikije gahunda yatanzwe cg wahawe?<br>**PDC; if necessary please explain PDC as: Gahunda ikorerwa mu bigo by'ubuvuzi yo gukurikirana no kwita ku mikurire y'abana bavukanye ibibazo by'ubuzima*	0 Oya<br>1 Yego<br>99 Simbizi
pdc_stop *(required)*	Kuki utakijyana umwana wawe muri gahunda ya PDC*?<br>**PDC; if necessary please explain PDC as: Gahunda ikorerwa mu bigo by'ubuvuzi yo gukurikirana no kwita ku mikurire y'abana bavukanye ibibazo by'ubuzima*	
pdc1 *(required)*	Niki washimye muri gahunda ya PDC?	0 Ntacyo<br>1 Porogoramu ya PDC yafashije umwana wanjye kugira ubuzima bwiza<br>2 PDC yampuje n'abandi babyeyi b'abana bafite ibibazo by'uburwayi nk'ubw'umwana wanjye<br>3 Abaganga bakora muri PDC baramfashije cyane<br>4 Muri PDC nahigiye uko nakwita ku mwana wanjye<br>5 PDC yahaye ibiribwa umuryango wanjye<br>6 Baduha amafaranga y'urugendo<br>20 Ikindi
pdc1_other *(required)*	Ubundi, sobanura:	
pdc2 *(required)*	Gahunda ya PDC wayiha amanota angahe ku birebana n'ireme rya service?	4 Byiza cyane/bihebuje<br>3 Byiza<br>2 Murugero<br>1 Munsi y'urugero<br>0 Bibi<br>99 Simbizi
pdc3 *(required)*	Ni izihe mbogamizi wahuye nazo mu kwitabira gahunda ya PDC?	1 Igihe cyo gutegereza ni kirekire<br>2 Hari abandi bana ntashobora gusiga mu rugo bonyine<br>3 Nta mpinduka mbona ku miterere y' uburwayi bw'umwana wanjye<br>4 Umufasha wanjye nta gaciro aha serivisi za porogaramu ya PDC<br>5 Serivise zirahenze<br>6 Serivise nta reme zifite<br>7 Akato/ivangura(bishobora gutuma bumva batahishyikira)<br>8 Ni uko umwana akiri muto cyane<br>9 Ibikorwa biri kure<br>10 Imiterere y'uburwayi bw'umwana wanjye ntizigera imera neza<br>11 Umwana wanjye nta kibazo cy'uburwayi afite bityo ntabwo akeneye kujyanwa ku kigo nderabuzima<br>12 Nta gaciro mpa gahunda ya PDC ku mwana wanjye<br>20 Izindi
pdc3_other *(required)*	Ubundi, sobanura:	
pdc4 *(required)*	Ni kuruhe rugero wishimye gahunda ya PDC?	0 Byaranyuze<br>1 Nta kidasanzwe<br>2 Ntibyanyuze<br>99 Simbizi
pdc5 *(required)*	Ese wagira inama ababyeyi cg abana kwitabira gahunda ya PDC?	0 Oya<br>1 Yego<br>99 Simbizi

Field	Question	Answer
pdc6 *(required)*	Niba ari yego, kubera iki wabagira iyo nama?	0 Ntacyo<br>1 Porogoramu ya PDC yafashije umwana wanjye kugira ubuzima bwiza<br>2 PDC yampuje n'abandi babyeyi b'abana bafite ibibazo by'uburwayi nk'ubw'umwana wanjye<br>3 Abaganga bakora muri PDC baramfashije cyane<br>4 Muri PDC nahigiye uko nakwita ku mwana wanjye<br>5 PDC yahaye ibiribwa umuryango wanjye<br>6 Baduha amafaranga y'urugendo<br>20 Ikindi
pdc6_other *(required)*	Ubundi, sobanura:	
Household Assets		
Ubu *(required)*	Ni ikihe cyiciro cy'ubudehe murimo?	1 Ubudehe 1<br>2 Ubudehe 2<br>3 Ubudehe 3<br>4 Ubudehe 4<br>7 Nta cyiciro<br>99 Simbizi<br>88 Nta gisubizo
WI5	Muri uru rugo mufite…	
WI6 *(required)*	Amashanyarazi?	0 Oya<br>1 Yego<br>99 Simbizi
WI7 *(required)*	Radio?	0 Oya<br>1 Yego<br>99 Simbizi
WI8 *(required)*	Telefone igendanwa?	0 Oya<br>1 Yego<br>99 Simbizi
WI9 *(required)*	Igare?	0 Oya<br>1 Yego<br>99 Simbizi
WI10 *(required)*	Ibyubatse hasi mu nzu. Andika ibyo ubona.	11 Umusenyi<br>12 Amase<br>21 Imbaho<br>22 Imikindo/ urugano<br>31 Imbaho zisennye<br>32 Vinyl or asphalt strips<br>33 Amakaro<br>34 Sima<br>35 Tapi/carpet<br>96 Ibindi
WI11 *(required)*	Ibindi, sobanura:	
WI12 *(required)*	Ni ibyumba bingahe muryamamo muri iyi nzu?	
WI13 *(required)*	Hari umuntu wo muri uru rugo woroye inka, inkoko cg andi matungo ye bwite?	0 Oya<br>1 Yego<br>99 Simbizi
cows *(required)*	Abantu bo mu rugo bafite inka zingahe?	
goats *(required)*	Mworoye ihene zingahe zanyu bwite?	
sheep *(required)*	Mworoye intama zingahe zanyu bwite?	
poultry *(required)*	Mworoye inkoko zingahe zanyu bwite?	
pigs *(required)*	Mworoye ingurube zingahe zanyu bwite?	
rabbits *(required)*	Mworoye inkwavu zingahe zanyu bwite?	
housing *(required)*	Ese muba mu nzu yanyu cyangwa murakodesha?	1 Inzu ni iyanjye/ni iy'uwo twashakanye<br>2 Inzu ni iy'undi muntu w'inshuti/ ni iy'umuvandimwe<br>3 Inzu irakodeshwa<br>6 Ibindi

Field	Question	Answer
own_land *(required)*	Ese urugo rwawe rufite isambu yo guhinga irimo ubwatsi bw'amatungo?	0 Oya<br>1 Yego<br>99 Simbizi
cook_place *(required)*	Akenshi mutekera hehe?	1 Mu nzu<br>2 Hanze y'inzu mu ruzitiro / mu gikari<br>3 Hanze y'inzu / mu mbuga / ku irembo<br>99 Simbizi
cook_sleep *(required)*	Ese wowe cg abana bawe urara / barara mu cyumba mutekeramo?	0 Oya<br>1 Yego<br>99 Simbizi
cook_fuel *(required)*	Ni iki mucana iyo muteka?	1 Gazi<br>2 Amashanyarazi<br>3 Inkwi<br>4 Amakara<br>5 Peteroli/Mazutu<br>6 Biyogazi<br>7 Ibishingwe/ibisigazwa by'ibihingwa cg ibisheshe<br>8 Ibindi<br>99 Simbizi
cook_fuel_other *(required)*	Vuga ubundi bwoko bw'ibicanwa	
Health Insurance		
hh_insured *(required)*	Haba hari umuntu wo muri uru rugo ufite ubwishingizi bw'ubuzima?	0 Oya<br>1 Yego<br>99 Simbizi
ins_type *(required)*	Ni ubuhe bwishingizi mu kwivuza mukoresha hano?	1 Mutuelle<br>2 RAMA<br>3 MMI<br>4 Ubwishingizi bwigenga (bwabikorera ku giti cyabo)<br>5 Ubundi
ins_type_other *(required)*	Ubundi, sobanura:	
all_insured *(required)*	Abagize uru rugo bose bivuriza kuri ubu bwishingizi?	1 Abagize urugo bose<br>2 Bamwe mu bagize uru rugo<br>99 Simbizi
child_insured *(required)*	Ese {child_name} afite ubwishingizi bwo kwivuza?	0 Oya<br>1 Yego<br>99 Simbizi
Household Savings		
savings1 *(required)*	Ese hari abantu bo mu muryango wawe bazigama amafaranga mu bigo by'imari byemewe n'amategeko (urugero nko muri SACCO, banki y'ubucuruzi)?	0 Oya<br>1 Yego<br>99 Simbizi
savings2 *(required)*	Ugereranije mu urugo rwanyu mubitsa amafaranga angahe buri kwezi muri SACCO cg banki?<br>*Enter as 1,000s of RWF, enter "99" if she/he does not know.*	
savings3 *(required)*	Ese hari abantu bo mu rugo rwawe bari mu kibina?	0 Oya<br>1 Yego<br>99 Simbizi
savings4 *(required)*	Ugereranije murugo rwanyu mubitsa amafaranga angahe buri kwezi mu kibina?<br>*Enter as 1,000s of RWF, enter "99" if she/he does not know.*	
Medical Expenses		
health_expend *(required)*	Ese mu mezi 3 ashize hari amafaranga waba warishyuye wivuza (amafaranga ashobora kuba yarishyuwe ako kanya/cash cg mu nguzanyo)?	0 Oya<br>1 Yego<br>99 Simbizi
health_expend2 *(required)*	Wishyuye amafaranga angahe? (ubariyemo n'inguzanyo)<br>*ENTER "1234" if amount NOT known.*	

Field	Question	Answer
health_expend3 *(required)*	Wishyuye iki?	1 Amafaranga yo kwisuzumisha ku ivuriro<br>2 Amafaranga yo kwisuzumisha k' umujyanama w'ubuzima<br>3 Amafaranga yishyurwa umuvuzi gakondo<br>4 Imiti<br>5 Ibikoresho(uturindantoki, ifishi, udukombe/uducupa bafatiramo ibizami kwa muganga, nibindi)<br>6 Ibizami byo muri laboratwari<br>7 Amafaranga yishyurwa n'ubwishingizi<br>8 Ay'urugendo<br>10 Ibindi<br>99 Simbizi
health_expend3a *(required)*	Ibindi, sobanura:	
pdc_expend *(required)*	Bigutwara amafaranga angahe iyo ugiye muri PDC*? Ubariyemo amafaranga wishyura y'urugendo, ayo wishyura kwa muganga, ay'imiti, ndetse n'andi yose wishyura ku bijyanye na gahunda ya PDC*.<br>*ENTER "1234" if amount NOT known.<br><br>*PDC; if necessary please explain PDC as: Gahunda ikorerwa mu bigo by'ubuvuzi yo gukurikirana no kwita ku mikurire y'abana bavukanye ibibazo by'ubuzima*	
Decision Making		
dm_note	Mu muryango wanyu, ninde ufata icyemezo cya nyuma ku buzima bwa {child_name}?	
dm_childsick *(required)*	Nko guhitamo icyakorwa igihe umwana arwaye	0 Se<br>1 Nyina<br>2 Ababyeyi bombi<br>3 Sogokuru<br>4 Nyogokuru<br>5 Abandi<br>99 Simbizi
dm_childeat *(required)*	Nko guhitamo ibyo umwana arya	0 Se<br>1 Nyina<br>2 Ababyeyi bombi<br>3 Sogokuru<br>4 Nyogokuru<br>5 Abandi<br>99 Simbizi
dm_money *(required)*	Ufata icyemezo mu ikoreshwa ry'amafaranga/icyo amafaranga yakoreshwa	0 Se<br>1 Nyina<br>2 Ababyeyi bombi<br>3 Sogokuru<br>4 Nyogokuru<br>5 Abandi<br>99 Simbizi
WASH		
WI1 *(required)*	Akenshi, amazi anyobwa n'abo muri uru rugo muyakura he?	11 Amazi ya robine iri mu nzu<br>12 Iri mu rugo/mu kibanza<br>13 Robine rusange<br>21 Amazi azamurwa na pompe cg itiyo<br>31 Iriba ritwikiriye<br>32 Ridatwikiriye<br>41 Amazi y'isoko yubakiye<br>42 Isoko itubakiye<br>51 Amazi y'imvura<br>61 Amazi azanwa n'ikamyo ya tanki<br>71 Baheka ku ngorofani nini ikururwa n'inka/n'indogobe<br>81 Amazi y'imigezi, ibiyaga, imiringoti, yo kuhira imirima<br>91 Amazi bapfundikira mu macupa<br>96 Ahandi
WI2 *(required)*	Ahandi, sobanura:	

Field	Question	Answer
water_insuf1 *(required)*	Mu byumweru bibiri bishize, hari igihe mu rugo rwanyu mutari mufite amazi ahagije kandi muyakeneye?	0 Oya<br>1 Yego<br>99 Simbizi
WI3 *(required)*	Umusarane mukoresha uteye ute?	11 Imisarane irekura amazi/ umusarane urekura amazi upfundikiye ufite fosse septique na puit perdu<br>12 Umusarane upfundikiye wohereza amazi mu itanki yabugenewe<br>13 Umusarane upfundikiye wohereza amazi mu cyobo cyabugenewe<br>14 Umusarani wohereza imyanda ahandi<br>15 Umusarani wohereza imyanda ahandi hatazwi<br>21 Umusarane utarekura amazi/ umusarani utinze neza ufite ubuhumekero<br>22 Umusarani utinze neza ufite aho bahagarara habugenewe<br>23 Umusarani w'icyobo kirangaye (udatinze)<br>31 Umusarani-ngarani<br>41 Kwituma mu gikoresho nk'indobo<br>51 Umusarani wubatse hejuru y'amazi (umena mu mazi)<br>61 Mu gisambu/ ku gasozi<br>96 Ahandi
WI4 *(required)*	Ahandi, sobanura:	
WS10 *(required)*	Twifuzaga kureba aho abo muri uru rugo bakarabira intoki.<br><br>Mushobora se ku twereka aho abagize umuryango wanyu bakunda gukarabira intoki?<br>*If there is not a designated place for handwashing, but instead a movable object like a basin, jerry can, or bucket select "Bucket, basin, or jerry can". If it is another object, please select "Other" and record response*	1 Wabibonye (fixed place for washing - sink, tippy tap)<br>4 Wabibonye: indobo, ibase, cg injerekani<br>2 ntabyo nabonye, Ntabiri mu urugo<br>3 Nta burenganzira bwo kubireba<br>5 Ibindi, sobanura:
WS11 *(required)*	Ibindi, sobanura:	
WS12 *(required)*	OBSERVATION.<br><br>Itegereze niba hari amazi aho bakarabira intoki.<br><br>Genzura niba hari robine, ibase, indobo, ikigega cy'amazi cyangwa ikindi kintu kirimo amazi.<br>*If there is a bucket, basin or other type of water container, examine to see whether water is present in the container. If you do not see water at the specific place for handwashing, code it as "Water not available"/"Ntamazi ahari"*	0 Yego, amazi arahari<br>1 Ntamazi ahari
WS13a *(required)*	Mwaba mufite isabune cyangwa imiti yica udukoko cyangwa ivu mukoresha mukaraba mu intoki hano mu rugo?	0 Oya<br>1 Yego<br>99 Simbizi
WS13b *(required)*	Ese hari isabune, imiti bakaraba mu ntoki igaragara aho bakarabira intoki?<br><br>Andika ibyo wabonye.<br><br>Reba aho bishobora gukoreshwa hose.	0 Ntayo<br>1 isabune irahari<br>2 imiti yica udukoko<br>3 isabune y'amazi<br>4 Ivu rirahari
Social Support for Caregiver		
issb_note	Ibibazo bikurikira ni ibibazo byerekeranye n'ubufasha ushobora kuba wahabwa n'abandi bantu mu buzima bwawe. Rimwe na rimwe abantu bagana abandi babashakaho ubufasha butandukanye. Ese mu kwezi gushize wahawe ubufasha butandukanye inshuro zingahe? Wibuke ko ibibazo ngiye kukubaza bijyanye n'ibyakubayeho mu kwezi gushize gusa.	
issb1 *(required)*	Ni kangahe waba warabonye abagusigarira ku rugo bita ku bo mubana cg ku bintu byawe igihe utari uhari?	1 Nta na rimwe<br>2 Gake<br>3 Rimwe na rimwe<br>4 Kenshi<br>5 Buri gihe<br>99 Simbizi

Field	Question	Answer
issb2 *(required)*	Ni kangahe ubona umuntu ukuba hafi igihe wumva ubabaye cg wigunze?	1 Nta na rimwe<br>2 Gake<br>3 Rimwe na rimwe<br>4 Kenshi<br>5 Buri gihe<br>99 Simbizi
issb3 *(required)*	Ni kangahe umuntu akubwira uko yitwaye mugihe yari afite ibibazo bisa nibyo ufite ubu?	1 Nta na rimwe<br>2 Gake<br>3 Rimwe na rimwe<br>4 Kenshi<br>5 Buri gihe<br>99 Simbizi
issb4 *(required)*	Ni kangahe umuntu akujyana mu bintu bishimishije bigufasha kwiyibagiza ibibazo byawe?	1 Nta na rimwe<br>2 Gake<br>3 Rimwe na rimwe<br>4 Kenshi<br>5 Buri gihe<br>99 Simbizi
issb5 *(required)*	Ni kangahe umuntu agufasha kubona uburyo wabaho neza?	1 Nta na rimwe<br>2 Gake<br>3 Rimwe na rimwe<br>4 Kenshi<br>5 Buri gihe<br>99 Simbizi
issb6 *(required)*	Ni kangahe umuntu agushima cg akuvuga neza ku byo wakoze?	1 Nta na rimwe<br>2 Gake<br>3 Rimwe na rimwe<br>4 Kenshi<br>5 Buri gihe<br>99 Simbizi
issb7 *(required)*	Ni kangahe umuntu yaguhaye amakuru ajyanye n'aho wajya bakagufasha?	1 Nta na rimwe<br>2 Gake<br>3 Rimwe na rimwe<br>4 Kenshi<br>5 Buri gihe<br>99 Simbizi
issb8 *(required)*	Ni kangahe umuntu aguhumuriza cg akubwira ko ibibazo bizashira?	1 Nta na rimwe<br>2 Gake<br>3 Rimwe na rimwe<br>4 Kenshi<br>5 Buri gihe<br>99 Simbizi
issb9 *(required)*	Ni kangahe umuntu agufasha kubona icyerekezo cy'ubuzima bwawe no gufata imigambi y'ejo hazaza?	1 Nta na rimwe<br>2 Gake<br>3 Rimwe na rimwe<br>4 Kenshi<br>5 Buri gihe<br>99 Simbizi
issb10 *(required)*	Ni kangahe umuntu akugira inama mu rwego rwo kugufasha mu bihe runaka?	1 Nta na rimwe<br>2 Gake<br>3 Rimwe na rimwe<br>4 Kenshi<br>5 Buri gihe<br>99 Simbizi
issb11 *(required)*	Ni kangahe ushobora kubona umuntu akuguriza/akaguha nibura amafranga igihumbi(1000fr) igihe uyakeneye?	1 Nta na rimwe<br>2 Gake<br>3 Rimwe na rimwe<br>4 Kenshi<br>5 Buri gihe<br>99 Simbizi
issb12 *(required)*	Ni kangahe ubona umuntu ugutega amatwi igihe uvuga uko wumva umerewe?	1 Nta na rimwe<br>2 Gake<br>3 Rimwe na rimwe<br>4 Kenshi<br>5 Buri gihe<br>99 Simbizi

Field	Question	Answer
issb13 *(required)*	Ni kangahe umuntu agutiza ibintu wari ukeneye (bitari amafranga)?	1 Nta na rimwe<br>2 Gake<br>3 Rimwe na rimwe<br>4 Kenshi<br>5 Buri gihe<br>99 Simbizi
issb14 *(required)*	Ni kangahe ubona umuntu ukungura ubumenyi ku kintu runaka ukeneye kumenya?	1 Nta na rimwe<br>2 Gake<br>3 Rimwe na rimwe<br>4 Kenshi<br>5 Buri gihe<br>99 Simbizi
issb15 *(required)*	Ni kangahe umuntu agusetsa kugira ngo akugarurire morale?	1 Nta na rimwe<br>2 Gake<br>3 Rimwe na rimwe<br>4 Kenshi<br>5 Buri gihe<br>99 Simbizi
issb16 *(required)*	Ni kangahe umuntu yaba yifatanya nawe gusenga?	1 Nta na rimwe<br>2 Gake<br>3 Rimwe na rimwe<br>4 Kenshi<br>5 Buri gihe<br>99 Simbizi
issb17 *(required)*	Ni kangahe umuntu akwitaho urwaye akakujyana kwa muganga cg ku muvuzi wa gakondo/gihanga igihe ubikeneye?	1 Nta na rimwe<br>2 Gake<br>3 Rimwe na rimwe<br>4 Kenshi<br>5 Buri gihe<br>99 Simbizi
issb18 *(required)*	Ni kangahe umuntu aza kugufasha gukora mu mirimo uba ukeneye ko ikorwa. Urugero: imirimo yo mu rugo,kuguhingira cg indi mirimo?	1 Nta na rimwe<br>2 Gake<br>3 Rimwe na rimwe<br>4 Kenshi<br>5 Buri gihe<br>99 Simbizi
issb19 *(required)*	Ni kangahe ubona umuntu ushobora kuguherekeza gusura abavandimwe bawe baba kure?	1 Nta na rimwe<br>2 Gake<br>3 Rimwe na rimwe<br>4 Kenshi<br>5 Buri gihe<br>99 Simbizi
issb20 *(required)*	Ni kangahe uba uri kumwe n'inshuti zawe?	1 Nta na rimwe<br>2 Gake<br>3 Rimwe na rimwe<br>4 Kenshi<br>5 Buri gihe<br>99 Simbizi
issb21 *(required)*	Ni kangahe ushobora kwiringira umuryango wawe n'inshuti zawe ngo zigufashe mu kibazo gikomeye?	1 Nta na rimwe<br>2 Gake<br>3 Rimwe na rimwe<br>4 Kenshi<br>5 Buri gihe<br>99 Simbizi
issb22 *(required)*	Ni kangahe wumva wisanzuye kubwira abantu impungenge zawe?	1 Nta na rimwe<br>2 Gake<br>3 Rimwe na rimwe<br>4 Kenshi<br>5 Buri gihe<br>99 Simbizi
issb23 *(required)*	Ni kangahe abantu bo mu muryango wawe n'inshuti zawe bagutegeka ibyo gukora?	1 Nta na rimwe<br>2 Gake<br>3 Rimwe na rimwe<br>4 Kenshi<br>5 Buri gihe<br>99 Simbizi

Field	Question	Answer
issb24 *(required)*	Ni kangahe umuntu yaba yaraguhaye ahantu ho kuba/icumbi?	1 Nta na rimwe<br>2 Gake<br>3 Rimwe na rimwe<br>4 Kenshi<br>5 Buri gihe<br>99 Simbizi
issb25 *(required)*	Ni kangahe umuntu akugira inama akagufasha kumva no gukemura ibibazo byawe?	1 Nta na rimwe<br>2 Gake<br>3 Rimwe na rimwe<br>4 Kenshi<br>5 Buri gihe<br>99 Simbizi
issb26 *(required)*	Ni kangahe umuntu agufasha uko ashoboye?	1 Nta na rimwe<br>2 Gake<br>3 Rimwe na rimwe<br>4 Kenshi<br>5 Buri gihe<br>99 Simbizi
issb27 *(required)*	Ni kangahe umuntu aguha ibintu by'ingenzi uba ukeneye?	1 Nta na rimwe<br>2 Gake<br>3 Rimwe na rimwe<br>4 Kenshi<br>5 Buri gihe<br>99 Simbizi
issb28 *(required)*	Ni kangahe umuntu akuganiriza iyo ubikeneye?	1 Nta na rimwe<br>2 Gake<br>3 Rimwe na rimwe<br>4 Kenshi<br>5 Buri gihe<br>99 Simbizi
issb30 *(required)*	Ni kangahe umuntu abahuriza hamwe ngo muganire ku bibazo muhuriyeho?	1 Nta na rimwe<br>2 Gake<br>3 Rimwe na rimwe<br>4 Kenshi<br>5 Buri gihe<br>99 Simbizi
issb32 *(required)*	Ni kangahe umuntu agufasha kuva mu bwigunge cg mu kwiheba?	1 Nta na rimwe<br>2 Gake<br>3 Rimwe na rimwe<br>4 Kenshi<br>5 Buri gihe<br>99 Simbizi
issb33 *(required)*	Ni kangahe umuntu akwereka urukundo?	1 Nta na rimwe<br>2 Gake<br>3 Rimwe na rimwe<br>4 Kenshi<br>5 Buri gihe<br>99 Simbizi
Medical Complications for the child		
MC2 *(required)*	Ese ubona umwana wawe afite amaso yeruruka, acika intege, cyangwa yaba yarigeze kongererwa amaraso?	0 Oya<br>1 Yego<br>99 Simbizi
MC3 *(required)*	Ese umwana wawe agira ibibazo mu mirire: nko kudatapfuna neza, ibibazo byo gukacanja cyangwa kumira, kunigwa n'ibiryo igihe arya?	0 Oya<br>1 Yego<br>99 Simbizi
MC3_duration *(required)*	Ese icyo kibazo cyatangiye afite mezi angahe?<br>*Enter response in months. Enter "99" if they do not know.*	
MC4 *(required)*	Ese umwana wawe ajya agira ibibazo byo guhumeka buri gihe nko guhumeka vuba na vuba, inkorora ihoraho, cyangwa kubura umwuka arimo kugenda?	0 Oya<br>1 Yego<br>99 Simbizi
MC5 *(required)*	Ese umwana wawe yari yigera ahwera?	0 Oya<br>1 Yego<br>99 Simbizi
MC5_b *(required)*	Ese umwana wawe ubu yaba agihwera?	0 Oya<br>1 Yego<br>99 Simbizi

Field	Question	Answer	
MC5_c *(required)*	Ese umwana wawe yaba muri iki gihe afata imiti imuvura guhwera, nka depakine cyangwa phenotoin?	0	Oya
		1	Yego
		99	Simbizi
readmit *(required)*	Ese umwana wawe yigeze asubizwa mu bitaro nyuma yo gusezererwa mu ishami ry'abana bavutse badashyitse?	0	Oya
		1	Yego
		99	Simbizi
readmit_freq *(required)*	Umwana wawe yashyizwe mu bitaro inshuro zingahe, hatarimo inshuro ya mbere yitabwaho mu ishami ry'abana bavutse badashyitse?		
readmit_when *(required)*	Ni ryari umwana wawe yasubijwe mu bitaro bwa mbere?	1	Mu kwezi 1 nyuma yo gusezererwa kwa muganga
		2	Mu mezi 2-5 nyuma yo kuva kwa muganga
		3	Urengeje amezi 6 nyuma yo kuva kwa muganga
		99	Simbizi
Infant and young child feeding practices			
iycf1 *(required)*	Ese {child_name} yaba yarigeze yonswa?	0	Oya
		1	Yego
		99	Simbizi
iycf2 *(required)*	Ese {child_name} aracyonswa?	0	Oya
		1	Yego
		99	Simbizi
diff_bf *(required)*	Wigeze uhura n'ibibazo byo konsa?	0	Oya
		1	Yego
		99	Simbizi
diff_bf_spec *(required)*	Ni ubuhe bwoko bw'ibibazo wahuye nabyo mu gihe cyo konsa?	1	Umubyeyi yatekerezaga ko afite amashereka make
		2	Kubabara cyangwa ibindi bibazo birebana n'ibere
		3	Nta gihe gihagije cyo konsa
		4	Afite ibibazo byo gufata ibere
		5	Afite ibibazo byo kumira
		6	Ikindi (sobanura)
		99	Simbizi
diff_bf_other *(required)*	Garagaza ubundi bwoko bw'ibibazo wahuye na byo		
diff_bf_help_yn *(required)*	Waba warabonye ubufasha mu gihe wari ufite ikibazo cyo konsa?	0	Oya
		1	Yego
		99	Simbizi
diff_bf_help *(required)*	Ni hehe waba warabonye ubufasha mu gihe wari ufite ikibazo cyo konsa?	1	Inshuti/abavandimwe
		2	Abavuzi ba gakondo
		3	ibitaro byo kurwego rw'igihugu
		4	Ku bitaro by'Akarere
		5	Ku igo nderabuzima
		6	Kuri post de sante
		7	Abajyanama b'ubuzima
		8	Imiryango itegamiye kuri leta (Save the Children, Caritas, Partners, cg ibindi)
		9	Abihayimana
		10	Ahandi
		11	Simbizi
infant_formula *(required)*	Wigeze ukoresha imfashabere z'abana mu kugaburira umwana?	0	Oya
		1	Yego
		99	Simbizi
formula_age *(required)*	Watangiye kumuha amata y'imfashabere ku mezi angahe?<br>*Enter age in months*		
formula_source *(required)*	Wakuye he amata y'imfashabere?	1	Abikorera ku giti cyabo (isoko)
		2	Imiryango itegamiye kuri leta (Save the Children, Caritas, Partners, cg ibindi)
		3	Ibigo by'ubuvuzi
		4	Leta
		6	Ikindi (sobanura)
		99	Simbizi
formula_source_other *(required)*	Vuga ahandi hantu hava imfashabere		

Field	Question	Answer
start_compfeed *(required)*	Yari afite imyaka ingahe mu gihe watangiraga kumuha ibindi binyobwa cyangwa ibiribwa bitari amashereka cyangwa imfashabere y'abana?	0 Umwana uri munsi y'ukwezi 1<br>1 Umwana w'ukwezi 1<br>2 Umwana w'amezi 2<br>3 Umwana w'amezi 3<br>4 Umwana w'amezi 4<br>5 Umwana w'amezi 5<br>6 Umwana w'amezi 6<br>7 Umwana w'amezi 7<br>8 Umwana w'amezi 8<br>9 Umwana w'amezi 9<br>10 Umwana w'amezi 10 cyangwa arenga<br>99 Simbizi
iycf36 *(required)*	Kuki mwatangiye guha {child_name} ibyo kurya n'ibyo kunywa bindi bitari amashereka (imfashabere)?<br>*Can select more than one response*	1 Yagiriwe inama n'uwo bashakanye<br>2 Nagiriwe inama na mama cg Mabukwe<br>3 Nagiriwe inama n'umujyanama w'ubuzima<br>4 (IZINA RY'UMWANA) yari ananutse<br>5 (IZINA RY'UMWANA) yarashonje kandi arira<br>6 Umubyeyi yagombagaga gukora kandi ntiyari ashoboye konsa umwana bihagije(Gukora birimo nko guhinga no kujya ku isoko)<br>7 umubyeyi yatekerezaga ko afite amashereka make<br>8 kubera ko umwana yari agejeje igihe cyo gutangira kurya.<br>9 Umubyeyi ntaba mu urugo<br>10 ikindi<br>99 Simbizi
Infant and young child's feeding practices: checklist of liquids		
iycf6	Ubu rero ndifuza kukubaza ku (ibindi) byo kunywa {child_name} yafashe ejo hashize kuva mu gitondo abyutse kugera nijoro agiye kuryama. Ndashaka kumenya niba yarafashe bimwe mubyo ngiye kuvuga kabone niyo yaba yarabifatanye n'ibindi biribwa.<br><br>Mushobora no gushyiramo ibyo kunywa yanyoye mu gasozi.<br><br>Ese {child_name} yaba yaranyoye (izina ry'ikintu) ejo, kumanywa cyangwa nijoro?	
iycf7 *(required)*	Amazi?	0 Oya<br>1 Yego<br>99 Simbizi
iycf8 *(required)*	Umutobe w'ubwoko ubwo aribwo bwose?	0 Oya<br>1 Yego<br>99 Simbizi
icyf_soda *(required)*	Fanta cg coca?	0 Oya<br>1 Yego<br>99 Simbizi
iycf10 *(required)*	Amata nk'ayo mu dusashe, ay'ifu cg amata yakamwe ku matungo/ inshyushyu cg ikivuguto?	0 Oya<br>1 Yego<br>99 Simbizi
iycf14 *(required)*	Ni inshuro zingahe {child_name} yanyweye amata?<br>*Niba ari inshuro 7 cg zirenga, andika 7. Niba ari simbizi, andika 99".*	
Dietary Diversity for Children 24+ mos		
dietdiv_instr	Ubu rero ndifuza kukubaza ku byo kurya {child_name} ashobora kuba yarafashe ejo kuva mu gitondo abyutse kugera nijoro agiye kuryama. Nanone, ndashaka kumenya niba yarafashe bimwe mubyo ngiye kuvuga kabone niyo yaba yarabifatanye n'ibindi biribwa.<br><br>Mushobora no gushyiramo ibyokurya yariye mugasozi.<br><br>Ese {child_name} yaba yarariiye (izina ry'ikintu) ejo ku manywa cyangwa nijoro?	

Field	Question	Answer
dietdiv1 *(required)*	IBINYAMPEKE: Ibigori, umuceri, ingano, amasaka cyangwa ibindi biribwa bikomoka ku binyampeke (urugero: umugati, makaroni, igikoma cg kawunga)	0 Oya<br>1 Yego<br>99 Simbizi
dietdiv2 *(required)*	IMBOGA N'IBINYABIJUMBA BIKUNGAHAYE KURI VITAMIN A: Ibihaza/Idegede/Imyungu, karoti, ibijumba by'umuhondo imbere, cyangwa izindi mboga zikungahaye kuri vitamin A?	0 Oya<br>1 Yego<br>99 Simbizi
dietdiv3 *(required)*	IMBOGA RWATSI ZIRIBWA IBIBABI: umushogoro, dodo, epinari, isombe, sukuma wiki, isogo, ibisusa?	0 Oya<br>1 Yego<br>99 Simbizi
dietdiv4 *(required)*	IZINDI MBOGA: ibitunguru, inyanya, amashu, intoryi, poivron, aubergine, beterave, cg izindi imboga	0 Oya<br>1 Yego<br>99 Simbizi
dietdiv5 *(required)*	IMBUTO VITAMIN A: Imyembe yeze, amapapayi, ibinyomoro, marakuja cyangwa ubundi bwoko bw'imbuto zikungahaye muri vitamin A?	0 Oya<br>1 Yego<br>99 Simbizi
dietdiv6 *(required)*	IZINDI IMBUTO: wotameroni, imineke, inanasi, avoka, amapera cg izindi imbuto	0 Oya<br>1 Yego<br>99 Simbizi
dietdiv7 *(required)*	IBINYABIJUMBA BY'UMWERU: Ibirayi, amateke, imyumbati, ibitoki cg ibindi bikomoka ku biribwa by'ibinyabijumba by'umweru.	0 Oya<br>1 Yego<br>99 Simbizi
dietdiv10 *(required)*	Inyama z'umwijima, impyiko, umutima, cyangwa izindi zo mu nda?	0 Oya<br>1 Yego<br>99 Simbizi
dietdiv11 *(required)*	INYAMA Z'UMUBIRI: nk'iz'inka, ingurube, intama, ihene, inkoko, cg imbata/dindon?	0 Oya<br>1 Yego<br>99 Simbizi
dietdiv12 *(required)*	Amagi? (ayariyo yose)	0 Oya<br>1 Yego<br>99 Simbizi
dietdiv13 *(required)*	IFI: Ifi itetse ikiva mu mazi/ikiri mbisi cyangwa yumye, isambaza, indagara, cyangwa saladini	0 Oya<br>1 Yego<br>99 Simbizi
dietdiv14 *(required)*	IMBOGA, UBUNYOBWA: Ibishyimbo, amashaza, lentiye, ibinyamavuta/ubunyobwa, soya n'ibindi biribwa bikoze muri ubwo bwoko by'ibyo tubabwiye?	0 Oya<br>1 Yego<br>99 Simbizi
dietdiv15 *(required)*	AMATA CG IBINDI BIKOZWE MU MATA: Foromage, ikivuguto, inshyushyu, cg ibindi bikoze mumata?	0 Oya<br>1 Yego<br>99 Simbizi
dietdiv16 *(required)*	IBINYAMAVUTA: Amamesa, ubuto, amavuta y'inka, amavuta y'ingurube (sendo)	0 Oya<br>1 Yego<br>99 Simbizi
dietdiv17 *(required)*	IBINYAMUSUKARI: isukali, ubuki, fanta, imitobe, jus, ibiryo biryohera nka shokola, bombo, biswi, mandazi, keke, icyayi cyangwa ikawa kirimo isukari	0 Oya<br>1 Yego<br>99 Simbizi
dietdiv18 *(required)*	Ibirungo bongera mu biryo n'ibyo bategura ku meza n'ibinyobwa: ibirungo (umunyu, magi, urusenda, akabanga) cg ibinyobwa (ikawa cyangwa icyayi kitarimo isukari, inzoga)	0 Oya<br>1 Yego<br>99 Simbizi
dietdiv19 *(required)*	Ejo hashize, hari ifunguro iryo ari ryo ryose umwana yaba yarariye ahandi hatari mu rugo (ifunguro ryuzuye cg ryoroheje)?	0 Oya<br>1 Yego<br>99 Simbizi
Meal Frequency for Child		
meal_freq_child *(required)*	Ejo hashize, ku manywa cg nijoro {child_name}, Ni inshuro zingahe yariye ibiryo Byumye/bikomeye, Byume/bikomeye gahoro, cg Byoroshye (bitose)?	
meal_freq_adult *(required)*	Ejo, ni inshuro zingahe abantu bakuru bo muri uru rugo bariye?<br>*Enter "99" if the cargeiver does not know*	
Household food security		
d4	Mu minsi 7 ishize, hari ubwo urugo rwawe habuze ibyo kurya bihagije cg amafaranga ahagije yo kugura ibyo kurya, ni kangahe byabaye murugo rwawe…	
d5 *(required)*	Kwifashisha cg kwibanda ku biribwa bidakunzwe kandi bidahenze?<br>*Enter "99" if the cargeiver does not know*	
d6 *(required)*	Gusaba ibyo kurya, cg gusaba ubufasha Inshuti cg umuvandimwe?<br>*Enter "99" if the cargeiver does not know*	
d7 *(required)*	Ibyo kurya bidahagije mu gihe cy'amafunguro?<br>*Enter "99" if the cargeiver does not know*	
d8 *(required)*	Hari igihe abantu bakuru bigomwa kurya kugirango abana bato babone ibyo barya?<br>*Enter "99" if the cargeiver does not know*	

Field	Question	Answer
d9 *(required)*	Kugabanya umubare w'inshuro zo kurya ku munsi?<br>*Enter "99" if the cargeiver does not know*	
Months of Food Availability		
mahfp_screen *(required)*	Noneho nagira ngo nkubaze ibijyanye no kuboneka ku ibiryo mu rugo rwanyu mu mezi atandukanye agize umwaka. Mu gusubiza ibi bizazo, gerageza usubize amaso inyuma utekereze mu mezi 12 ashize, uhereye mu kwezi gushize uyu mwaka, usubiye inyuma ukageze muri uku kwezi umwaka ushize. Ese muri icyo gihe haba hari ukwezi mwagizemo ibibazo byo kubura ibitunga urugo bihagije cg amafaranga yo kubigura?	0 Oya<br>1 Yego<br>99 Simbizi
mahfp1 *(required)*	Niba ari yego, ni mu yahe mezi urugo rwawe rutari rufite amafaranga ahagije yo guhaha?	22 Kamena 2019<br>21 Gicurasi 2019<br>20 Mata 2019<br>19 Werurwe 2019<br>18 Gashyantare 2019<br>17 Mutarama 2019<br>16 Ukuboza 2018<br>15 Ugushyingo 2018<br>1 Ukwakira 2018<br>2 Nzeri 2018<br>3 Kanama 2018<br>4 Nyakanga 2018<br>5 Kamena 2018<br>6 Gicurasi 2018
mahfp2 *(required)*	Ese waba warahuye ni iki kibazo incuro nyinshi muri kiriya gihe mu rugo rwawe?	0 Oya<br>1 Yego<br>99 Simbizi
Caregiver Mental Health		
hscl_note	AMABWIRIZA: Ibi bikurikira ni ibimenyetso cyangwa ibibazo abantu bagira rimwe na rimwe. Ngiye kugusomera buri kibazo maze umbwire inshuro byaba byarakubayeho cyangwa se byaragutesheje umutwe mu cyumweru gishize, ubariyemo n'uyu munsi.	
hscl1 *(required)*	Gutahwa n'ubwoba nta mpamvu igaragara	0 Habe na gito<br>1 Gito<br>2 Kenshi<br>3 Buri Gihe<br>99 Simbizi
hscl2 *(required)*	Kumva utinya/ufite impungenge	0 Habe na gito<br>1 Gito<br>2 Kenshi<br>3 Buri Gihe<br>99 Simbizi
hscl3 *(required)*	Kugwa igihumure, kugira isereri, cyangwa se gucika intege	0 Habe na gito<br>1 Gito<br>2 Kenshi<br>3 Buri Gihe<br>99 Simbizi
hscl4 *(required)*	Guhangayika cyangwa se kumva ushegeshwe	0 Habe na gito<br>1 Gito<br>2 Kenshi<br>3 Buri Gihe<br>99 Simbizi
hscl5 *(required)*	Umutima utera cyane cyangwa se usimbuka	0 Habe na gito<br>1 Gito<br>2 Kenshi<br>3 Buri Gihe<br>99 Simbizi
hscl6 *(required)*	Gutitira	0 Habe na gito<br>1 Gito<br>2 Kenshi<br>3 Buri Gihe<br>99 Simbizi
hscl7 *(required)*	Kumva wifitememo icyoba	0 Habe na gito<br>1 Gito<br>2 Kenshi<br>3 Buri Gihe<br>99 Simbizi

Field	Question	Answer
hscl8 *(required)*	Umutwe	0 Habe na gito<br>1 Gito<br>2 Kenshi<br>3 Buri Gihe<br>99 Simbizi
hscl9 *(required)*	Kuvugana ubwoba cyangwa igihunga	0 Habe na gito<br>1 Gito<br>2 Kenshi<br>3 Buri Gihe<br>99 Simbizi
hscl10 *(required)*	Kumva udatuje, udashobora kwicara hamwe	0 Habe na gito<br>1 Gito<br>2 Kenshi<br>3 Buri Gihe<br>99 Simbizi
hscl11 *(required)*	Kumva wacitse intege	0 Habe na gito<br>1 Gito<br>2 Kenshi<br>3 Buri Gihe<br>99 Simbizi
hscl12 *(required)*	Kwishinja	0 Habe na gito<br>1 Gito<br>2 Kenshi<br>3 Buri Gihe<br>99 Simbizi
hscl13 *(required)*	Kurizwa n`ubusa	0 Habe na gito<br>1 Gito<br>2 Kenshi<br>3 Buri Gihe<br>99 Simbizi
hscl14 *(required)*	Kumva nta bushake bwo gukora imibonano mpuzabitsina cyangwa se kutayishimira	0 Habe na gito<br>1 Gito<br>2 Kenshi<br>3 Buri Gihe<br>99 Simbizi
hscl15 *(required)*	Kudashaka kurya (kutaryoherwa)	0 Habe na gito<br>1 Gito<br>2 Kenshi<br>3 Buri Gihe<br>99 Simbizi
hscl16 *(required)*	Birakugora kubona ibitotsi no gusinzira	0 Habe na gito<br>1 Gito<br>2 Kenshi<br>3 Buri Gihe<br>99 Simbizi
hscl17 *(required)*	Gutakaza ikizere cy'ejo hazaza	0 Habe na gito<br>1 Gito<br>2 Kenshi<br>3 Buri Gihe<br>99 Simbizi
hscl18 *(required)*	Kumva ubabaye	0 Habe na gito<br>1 Gito<br>2 Kenshi<br>3 Buri Gihe<br>99 Simbizi
hscl19 *(required)*	Kwigunga no kwiheza	0 Habe na gito<br>1 Gito<br>2 Kenshi<br>3 Buri Gihe<br>99 Simbizi
hscl20 *(required)*	Ibitekerezo biganisha ku kwiyambura ubuzima/kwiyahura<br>*[RISK OF HARM PROTOCOL QUESTION]. If participant responds "Kenshi" or "Buri Gihe", express concern about this and follow up to assess severity.*	0 Habe na gito<br>1 Gito<br>2 Kenshi<br>3 Buri Gihe<br>99 Simbizi

Field	Question	Answer
hscl21 *(required)*	Kumva ugoswe/ugeramiwe	0 Habe na gito<br>1 Gito<br>2 Kenshi<br>3 Buri Gihe<br>99 Simbizi
hscl22 *(required)*	Kumva uhangayitse cyane	0 Habe na gito<br>1 Gito<br>2 Kenshi<br>3 Buri Gihe<br>99 Simbizi
hscl23 *(required)*	Kumva nta kintu na kimwe kigushimishije cg uha agaciro	0 Habe na gito<br>1 Gito<br>2 Kenshi<br>3 Buri Gihe<br>99 Simbizi
hscl24 *(required)*	Kumva buri kintu cyose ari ukwihata	0 Habe na gito<br>1 Gito<br>2 Kenshi<br>3 Buri Gihe<br>99 Simbizi
hscl25 *(required)*	Kumva nta gaciro ufite	0 Habe na gito<br>1 Gito<br>2 Kenshi<br>3 Buri Gihe<br>99 Simbizi
Service Access		
ecd_attend *(required)*	{child_name} yaba yiga muri amwe mu mashuri y'incuke, ryaba iryigenga cg irya LETA cg aho barerera abana b'incuke cg aho basiga abana b'incuke?	0 Oya<br>1 Yego<br>99 Simbizi
ecd_hours *(required)*	Muminsi 7 ishize, ni amasaha angahe{child_name} yagiye aho hantu?	
growth_mon *(required)*	Mu kwezi gushize, {child_name} yaba yaragiye kwa muganga cg umujyanama w'ubuzima ngo bamupime imikurire ye?	0 Oya<br>1 Yego<br>99 Simbizi
maln_prog *(required)*	Ese umwana wawe yaba yarigeze kwandikwa muri gahunda runaka kubera imirire mibi?<br>*This DOES NOT INCLUDE services at PDC. We are referring to malnutrition programs at hospital or health centers.*	0 Oya<br>1 Yego<br>99 Simbizi
maln_prog_type *(required)*	Ni iyihe gahunda yerekeye imirere mibi umwana wawe yiyandikishijemo?	1 Gahunda y'ikigo nderabuzima yita kubazahajwe n'imirire mibi (OTP)<br>2 Gahunda y'ikigo nderabuzima yita kubafite imirire mibi bigereranyije (SFP)<br>3 Gahunda y'ikigo nderabuzima (ahandi)<br>4 Abagiye mu bitaro kubera imirire mibi<br>5 Gahunda zo kwigisha abaturage (gukurikirana imikurire y'umwana aho batuye, kwerekerwa uko babatekera)<br>10 Ikindi (sobanura)
maln_prog_current *(required)*	Ese ubu yanditse muri iyo gahunda?	0 Oya<br>1 Yego<br>99 Simbizi
maln_prog_time *(required)*	Ni ryari umwana wawe aheruka kwandikwa muri iyo gahunda?	1 . < amezi 6 ashize<br>2 mu mezi 6 kugeza ku mezi 11 ashize<br>3 >amezi 12(umwaka 1) ashize<br>10 Ikindi (sobanura)
maln_prog_time_other *(required)*	Andika igihe umwana aheruka kwandikwa<br>*Ideally month and year. Enter only year if caregiver is not sure of the month. Enter "99" if caregiver does not know.*	
Child Discipline		
cd_note	AMABWIRIZA: Abantu bakuru bakoresha uburyo butandukanye mu kwigisha abana imyitwarire iboneye cyangwa mu gukosora imibi. Ngiye kubasomera uburyo butandukanye bukoreshwa maze mbasabe kumbwira niba wowe ubwawe cyangwa se undi muntu mubana yaba yarabukoresheje mu minsi 30 ishize:	

Field	Question	Answer
cd1 *(required)*	Wowe cg undi muntu mubana yaba yaramwimye ibintu yashakaga kubera imyitwarire mibi	0 Oya<br>1 Yego<br>99 Simbizi
cd3 *(required)*	Wowe cg undi muntu mubana yaba yaramusobanuriye impamvu imyitwarire ye yari mibi.	0 Oya<br>1 Yego<br>99 Simbizi
cd4 *(required)*	Wowe cg undi muntu mubana yaba yaramushikanuje igihe yari yamurakariye	0 Oya<br>1 Yego<br>99 Simbizi
cd5 *(required)*	Wowe cg undi muntu mubana yaba yaramukankamiye, yaramututse cg yaramutombokanye	0 Oya<br>1 Yego<br>99 Simbizi
cd6 *(required)*	Wowe cg undi muntu mubana yaba yaramuhanishije gukora imirimo, kugirango adakomeza kugira imyitwarire mibi.	0 Oya<br>1 Yego<br>99 Simbizi
cd7 *(required)*	Wowe cg undi muntu mubana yaba yaramukubise inshyi ku kibuno	0 Oya<br>1 Yego<br>99 Simbizi
cd8 *(required)*	Wowe cg undi muntu mubana yaba yaramukubise ku kibuno cg ahandi hantu akoresheje umukandara, inkoni cg ikindi gikoresho gikomeye.	0 Oya<br>1 Yego<br>99 Simbizi
cd9 *(required)*	Wowe cg undi muntu mubana yaba yaramwise igicucu, umunebwe, ikigoryi, cg andi mazina mabi nk'ayo	0 Oya<br>1 Yego<br>99 Simbizi
cd10 *(required)*	Wowe cg undi muntu mubana yaba yaramukubise mu maso, mu mutwe cg mu matwi.	0 Oya<br>1 Yego<br>99 Simbizi
cd11 *(required)*	Wowe cg undi muntu mubana yaba yaramukubise ku kiganza, ku maboko cg ku maguru.	0 Oya<br>1 Yego<br>99 Simbizi
cd12 *(required)*	Wowe cg undi muntu mubana yaba yararamuhondaguye cyane.<br>*[RISK OF HARM PROTOCOL QUESTION]. This item indicates potential concern about abuse. If the caregiver says YES, express concern about this and follow up to assess severity.*	0 Oya<br>1 Yego<br>99 Simbizi
cd13 *(required)*	Utekereza ko kurera umwana, cg kumwigisha neza biba ngombwa ko umwana akubitwa?	0 Oya<br>1 Yego<br>99 Simbizi
ECD Index - MICS		
picture_books *(required)*	Ufite ibitabo b'ingahe by'abana cg ibitabo by'amashusho bya {child_name}?<br>*Enter "99" if caregiver does not know.*	
ecd_index_note	Ndashaka kumenya ubwoko bw'ibikinisho {child_name} akina iyo ari murugo.<br>*AMABWIRIZA: NIBA USUBIZA AGUSUBIJE "YEGO" KURI IBI BYAVUZWE HEJURU , MUSOBANUZE NEZA KUGIRANGO UMENYE NEZA IGIKINISHO UMWANA AKORESHA KIJYANYE NIGISUBIZO NYACYO.*	
homemade_toys *(required)*	Ibikinisho by' abana byakorewe mu rugo/ bitakorewe mu nganda (nk' bipupe, imodoka, cg ibindi bikinisho byakorewe murugo)?	0 Oya<br>1 Yego<br>99 Simbizi
purchased_toys *(required)*	Ibikinisho by'abana/ ibipupe byaguzwe mu iduka cg inganda zikora ibikinisho by' abana?	0 Oya<br>1 Yego<br>99 Simbizi
household_toys *(required)*	Ibikoresho byo murugo nka biye/bowls ibibumbano cg ibindi bikoresho biboneka (nk'inkoni, amabuye, impu z'ibikoko cg amababi, impu z'inyamaswa.	0 Oya<br>1 Yego<br>99 Simbizi
inad_alone *(required)*	N'iminsi ingahe mu cyumweru gishize {child_name} yaba yarasigaye murugo wenyine mu gihe kirenze isaha?<br>*If none enter 0.*	
inad_child *(required)*	N'iminsi ingahe mu cyumweru gishize {child_name} yaba yarasigaranye n'undi mwana uri munsi y'imyaka 10. Mugihe kirenze isaha?<br>*If none enter 0.*	
ECD Index - MICS		
ecd_index_note2	Mumunsi 3 ishize waba /undi muntu wo murugo ufite kuva ku myaka 15 gusubiza hejuru yarakoresheje imwe muri iyi mirimo/ibi bikorwa {child_name}:<br><br>*AMABWIRIZA: Niba ari yego, mubaze:<br>*Ninde wakoreshejeje/witabiriye iyi mirimo na {child_name}?<br><br>*CA AKAZIGA KUCYO AKUBWIYE.<br>**DO NOT READ RESPONSE OPTIONS OUT LOUD.*	

Field	Question	Answer
ecd_index1 *(required)*	Kusomera ibitabo cg kwereka amashusho/amafoto {child_name}?<br>*Select all that are mentioned.*	1 Mama/Nyina<br>2 Papa/Ise<br>3 Undi<br>4 Ntanumwe
ecd_index2 *(required)*	Kubarira inkuru {child_name}?<br>*Select all that are mentioned.*	1 Mama/Nyina<br>2 Papa/Ise<br>3 Undi<br>4 Ntanumwe
ecd_index3 *(required)*	Kuririmbira {child_name}, ushyiremo utubyiniriro?<br>*Select all that are mentioned.*	1 Mama/Nyina<br>2 Papa/Ise<br>3 Undi<br>4 Ntanumwe
ecd_index4 *(required)*	Gutembereza {child_name} hanze y'urugo, inzu, uruzitiro cg igipangu?<br>*Select all that are mentioned.*	1 Mama/Nyina<br>2 Papa/Ise<br>3 Undi<br>4 Ntanumwe
ecd_index5 *(required)*	Akinisha/akinana na {child_name}?<br>*Select all that are mentioned.*	1 Mama/Nyina<br>2 Papa/Ise<br>3 Undi<br>4 Ntanumwe
ecd_index6 *(required)*	Yigisha amazina y'ibintu, kubara, gushushanya?<br>*Select all that are mentioned.*	1 Mama/Nyina<br>2 Papa/Ise<br>3 Undi<br>4 Ntanumwe
LONG FORM: MOTOR, COGNITIVE, LANGUAGE, & SOCIAL-EMOTIONAL		
LF_note	Noneho ngiye ku kubaza ibintu umwana wawe ashobora gukora. Subiza "yego" cg "oya" kuri ibi bibazo bikurikira. Niba utabizi neza ushobora gusubiza "simbizi". Wibuke ko abana biga kandi bakura mu buryo butandukanye. Nta kibazo niba hari ibintu umwana wawe atarakora. Bimwe muri ibi bintu hari ibyo abana bakora batinze. Niba hari ikibazo udashaka gusubiza, urabimenyesha noneho tugisimbuke tujye ku bindi.	
LF1 *(required)*	Ese iyo umwana aryamye agaramye anyeganyeza amaboko n'amaguru bye?<br>*Start point for 0-5 months*	1 Yego<br>0 Oya<br>0.1 Simbizi
LF2 *(required)*	Ese ashyira akaboko ku munwa we?	1 Yego<br>0 Oya<br>0.1 Simbizi
LF3 *(required)*	Ese umwana araseka?	1 Yego<br>0 Oya<br>0.1 Simbizi
LF4 *(required)*	Ese umwana araseka iyo abonye abandi bamusekera?	1 Yego<br>0 Oya<br>0.1 Simbizi
LF5 *(required)*	Ese umwana yonka urutoki rw'igikumwe cg izindi?	1 Yego<br>0 Oya<br>0.1 Simbizi
LF6 *(required)*	Ese umwana afata ikintu gito (e.g: urutoki rwawe, ikiyiko) iyo ubishyize mu kiganza cye?<br>*Start point for 6-11 months*	1 Yego<br>0 Oya<br>0.1 Simbizi
LF7 *(required)*	Ese umwana ashobora gufatanya amaboko ye?	1 Yego<br>0 Oya<br>0.1 Simbizi
LF8 *(required)*	Ese umwana arakumenya cg abandi bantu bo mu muryango (e.g: araseka iyo binjiye mu nzu cg batambutse)?	1 Yego<br>0 Oya<br>0.1 Simbizi
LF9 *(required)*	Ese umwana wawe ahora afunze igipfunsi?	0 Yego<br>1 Oya<br>0.1 Simbizi
LF10 *(required)*	Ese iyo umwana bamushyize imbere ibintu bishya yerekana ko abyitayeho agashaka kubikoraho?	1 Yego<br>0 Oya<br>0.1 Simbizi
LF11 *(required)*	Umwana ashobora kwihindukiza imbere n'inyuma?<br>*Start point for 12-17 months*	1 Yego<br>0 Oya<br>0.1 Simbizi
LF12 *(required)*	Ese umwana yerekana ko yitaye ku bintu bishya abonye ashaka kubishyira mu kanwa?	1 Yego<br>0 Oya<br>0.1 Simbizi

Field	Question	Answer
LF13 *(required)*	Umwana yereka abandi urugwiro (urugero: ahobera ababyeyi, basaza be cg bashiki be?)	1 Yego<br>0 Oya<br>0.1 Simbizi
LF14 *(required)*	Ese umwana ashobora gufata ikintu (urugero: agakinisho gato cg akabuye gato), akoresheje ukuboko kumwe?	1 Yego<br>0 Oya<br>0.1 Simbizi
LF15 *(required)*	Umwana ajya ashakisha igikinisho cg ikindi kintu kimushishikaje iyo wagihishe cg wakivanye aho yakirebaga (urugero: wagipfutse cg wagihishe munsi y'ikindi kintu)?	1 Yego<br>0 Oya<br>0.1 Simbizi
LF16 *(required)*	Ese iyo umwana aryamye afata ibirenge bye?	1 Yego<br>0 Oya<br>0.1 Simbizi
LF17 *(required)*	Ese umwana abasha kuvuga amajwi nka ba, da cg do"?	1 Yego<br>0 Oya<br>0.1 Simbizi
LF18 *(required)*	Ashobora kuzamura umutwe, akoresheje amaboko gusa mu gihe aryamye yubitse inda?	1 Yego<br>0 Oya<br>0.1 Simbizi
LF19 *(required)*	Ese umwana ajya akina akubita ikintu ku butaka cg ku meza?	1 Yego<br>0 Oya<br>0.1 Simbizi
LF20 *(required)*	Ese umwana ashobora kwiyicaza akamara akanya nta muntu umufashe?	1 Yego<br>0 Oya<br>0.1 Simbizi
LF21 *(required)*	Ese umwana ajya agenda cg akava aho yari ari ku bushake ajya gufata ikintu kiri kure?	1 Yego<br>0 Oya<br>0.1 Simbizi
LF22 *(required)*	Ese umwana ashobora kureba ikintu umweretse mu gihe umuntu avuze ngo "reba" akagitunga urutoki?	1 Yego<br>0 Oya<br>0.1 Simbizi
LF23 *(required)*	Ese umwana azi izina rye bwite cg izina ry'akabyiniriro? Bisobanuye ko ibyo akora iyo umuhamagaye mu izina bitandukanye n'ibyo akora iyo yumvise andi magambo?	1 Yego<br>0 Oya<br>0.1 Simbizi
LF24 *(required)*	Iyo uvugishije umwana, agusubiza akoresha amajwi (urugero:"ba", "da", cg "do") cg akavuga ijambo?	1 Yego<br>0 Oya<br>0.1 Simbizi
LF25 *(required)*	Ese umwana ashobora gukambakamba, kwihindukiza cg gukambakamba agana imbere nta wumufashije?	1 Yego<br>0 Oya<br>0.1 Simbizi
LF26 *(required)*	Ese umwana ashobora gufata, akitamika ibiryo akoresheje intoki?	1 Yego<br>0 Oya<br>0.1 Simbizi
LF27 *(required)*	Ese umwana ashobora gukura ikintu gito (urugero: agakinisho gato cg akabuye) mu kaboko kamwe ashyira mu kundi kuboko?	1 Yego<br>0 Oya<br>0.1 Simbizi
LF28 *(required)*	Ese umwana abasha gukoma mu mashyi?	1 Yego<br>0 Oya<br>0.1 Simbizi
LF29 *(required)*	Ese umwana ashobora kuguma ahagaze afashe ku muntu cg ku kintu?<br>*Start point for 18-23 months*	1 Yego<br>0 Oya<br>0.1 Simbizi
LF30 *(required)*	Ese umwana wawe ashobora gukoresha amarenga agamije ku kwereka icyo ashaka (urugero: azamura akaboko yerekana ko ashaka ko bamuterura cg atunga urutoki amazi)?	1 Yego<br>0 Oya<br>0.1 Simbizi
LF31 *(required)*	Ashobora gukoresha igikumwe n'urutoki rukurikira igikumwe (urugero:agatora agakinisho gato cg akabuye gato)?	1 Yego<br>0 Oya<br>0.1 Simbizi
LF32 *(required)*	Ese umwana ashobora gufata cg kurekurira hasi ikintu gito (urugero: agakinisho gato cg akabuye) mu ndobo cg ibakure mu gihe yicaye?	1 Yego<br>0 Oya<br>0.1 Simbizi
LF33 *(required)*	Ese umwana wawe ashobora kujugunya umupira cg akabuye (yerekeza imbere) akoresheje ukuboko?	1 Yego<br>0 Oya<br>0.1 Simbizi

Field	Question	Answer
LF34 *(required)*	Ese umwana ashobora gutambuka intambwe nyinshi yifashishije amaboko cg ikindi kintu (urugero: urukuta cg intebe)?	1 Yego<br>0 Oya<br>0.1 Simbizi
LF35 *(required)*	Ese umwana wawe ashobora kuvuga ijambo rimwe cg menshi (urugero: amazina nka "mama" cg "pi" ashaka kuvuga "umupira")?	1 Yego<br>0 Oya<br>0.1 Simbizi
LF36 *(required)*	Ese umwana ashobora guhagarara nta kintu yifashishije	1 Yego<br>0 Oya<br>0.1 Simbizi
LF37 *(required)*	Ese umwana wawe ashobora gukurikira ibyerekezo bisanzwe (urugero: "haguruka" cg "ngwino hano")?	1 Yego<br>0 Oya<br>0.1 Simbizi
LF38 *(required)*	Ese umwana areba ibyo abandi bana bakora yarangiza akagerageza kubigana?	1 Yego<br>0 Oya<br>0.1 Simbizi
LF39 *(required)*	Ese umwana ashobora kwicara cg kwikinana nibura mu gihe cy'iminota 20?<br>*Start point for 24-29 months*	1 Yego<br>0 Oya<br>0.1 Simbizi
LF40 *(required)*	Umwana ashobora gutambuka intambwe nyinshi nta kintu yifashishije cg ngo ahabwe ubundi bufasha?	1 Yego<br>0 Oya<br>0.1 Simbizi
LF41 *(required)*	Umwana ashobora kunama hanyuma akunamuka ataguye kandi atifashishije umuntu cg ikintu?	1 Yego<br>0 Oya<br>0.1 Simbizi
LF42 *(required)*	Ese umwana wawe ajya agusaba ubufasha iyo ashaka ikintu akoresheje amarenga cg amagambo iyo adashobora kubyikorera (urugero: kugera ku kintu kiri kure)?	1 Yego<br>0 Oya<br>0.1 Simbizi
LF43 *(required)*	Umwana agerageza kwigana amajwi cg amagambo yavuzwe n'abandi bantu?	1 Yego<br>0 Oya<br>0.1 Simbizi
LF44 *(required)*	Ese umwana wawe ashobora kurira intebe cg urubaho?	1 Yego<br>0 Oya<br>0.1 Simbizi
LF45 *(required)*	Ashobora kubura ikiyiko cg ikindi kintu mu gihe wakimuhaye cyubitse cg gicuritse?	1 Yego<br>0 Oya<br>0.1 Simbizi
LF46 *(required)*	Iyo ubujije umwana gukora ikintu, arahagarara nibura akanya gato?	1 Yego<br>0 Oya<br>0.1 Simbizi
LF47 *(required)*	Ese umwana ashobora gutera umupira cg ikindi kintu kimeze nk'umupira mu cyerekezo cy'imbere?	1 Yego<br>0 Oya<br>0.1 Simbizi
LF48 *(required)*	Ese umwana ashobora gutunga urutoki umuntu cg ikintu niba ubimusabye (urugero:" mama arihe?" cg "umupira urihe")?	1 Yego<br>0 Oya<br>0.1 Simbizi
LF49 *(required)*	Ese umwana ashobora kunywesha icupa (ridapfundikiye) atabyimenyeho?	1 Yego<br>0 Oya<br>0.1 Simbizi
LF50 *(required)*	Umwana yigana amajwi y'ibikoko cg andi majwi (urugero: "vroom" y'imodoka "moo" y'inka)?	1 Yego<br>0 Oya<br>0.1 Simbizi
LF51 *(required)*	Ese umwana ashobora kwiruka intambwe nyinshi ataragwa cg ahitane ibintu?	1 Yego<br>0 Oya<br>0.1 Simbizi
LF52 *(required)*	Umwana ashobora guca umurongo cg agasharambura k'urupapuro n'ikaramu cg ikaramu y'igiti, cg ku butaka n'igiti?<br>*Start point for 30-35 months*	1 Yego<br>0 Oya<br>0.1 Simbizi
LF53 *(required)*	Ese umwana wawe ashobora gusubiza ibibazo bisanzwe (urugero: " urashaka amazi?") asubiza ngo "yego" cg "oya" aho kuzunguza umutwe?	1 Yego<br>0 Oya<br>0.1 Simbizi
LF54 *(required)*	Ese umwana wawe ashobora kugerekeranya ibintu 3 bito cg byinshi(urugero: amatafari, ibikombe, amacupa) hejuru y'ibindi?	1 Yego<br>0 Oya<br>0.1 Simbizi

Field	Question	Answer
LF55 *(required)*	Ese umwana yigana imico yabandi (urugero: gukaraba intoki cg koza ibyombo)?	1 Yego<br>0 Oya<br>0.1 Simbizi
LF56 *(required)*	Rimwe na rimwe umwana ajya yibwiriza agasangira n'abandi (urugero:ibiryo, ibikinisho)?	1 Yego<br>0 Oya<br>0.1 Simbizi
LF57 *(required)*	Umwana ashobora gukurikiza amabwiriza cg amategeko atandukanye(Urugero: jya kuzana amazi n'urangiza ujye kuryama")?	1 Yego<br>0 Oya<br>0.1 Simbizi
LF58 *(required)*	Ese umwana ashobora kuvuga amagambo 5 cg arenga (Urugero: amazina nka "mama" cg" ibintu nk'umupira)?	1 Yego<br>0 Oya<br>0.1 Simbizi
LF59 *(required)*	Ese umwana akunda abana bato (Urugero: abavugisha neza akanabakoraho gahoro gahoro)?	1 Yego<br>0 Oya<br>0.1 Simbizi
LF60 *(required)*	Umwana ashobora kugenda mu mihanda mibi (Urugero: irimo imikuku) ataguye?	1 Yego<br>0 Oya<br>0.1 Simbizi
LF61 *(required)*	Umwana ntarangara iyo barimo kumubarira inkuru?	1 Yego<br>0 Oya<br>0.1 Simbizi
LF62 *(required)*	Ese umwana ashobora gusaba (Urugero: ibiryo, amazi) mu mazina yabyo mugihe abikeneye?	1 Yego<br>0 Oya<br>0.1 Simbizi
LF63 *(required)*	Ese umwana ajya akinana n'abandi bana (Urugero: imikino akinana n'abandi bana)?	1 Yego<br>0 Oya<br>0.1 Simbizi
LF64 *(required)*	Ese umwana ashobora kumenya nibura izina ry'undi muntu wo mu muryango atari mama na papa (Urugero: izina rya musaza we, mushikiwe we, nyirasenge, nyirarume)?	1 Yego<br>0 Oya<br>0.1 Simbizi
LF65 *(required)*	Ese umwana ajya akinisha ibintu abyita ibindi (Urugero: atekereza ko icupa ari igipupe, ibuye ari imodoka, cg ikiyiko ari indege)?	1 Yego<br>0 Oya<br>0.1 Simbizi
LF66 *(required)*	Ese umwana ajya yerekana ko ababaye cg agahangayikishwa nuko abandi barakaye cg bababaye?	1 Yego<br>0 Oya<br>0.1 Simbizi
LF67 *(required)*	Ese umwana ashobora kugenda asubira inyuma?	1 Yego<br>0 Oya<br>0.1 Simbizi
LF68 *(required)*	Ese umwana afite amatsiko yo kwiga ibintu bishya (Urugero: abaza ibibazo cg kumenya ibintu bishya)?	1 Yego<br>0 Oya<br>0.1 Simbizi
LF69 *(required)*	Umwana ashobora kwirisha akoresheje ikiyiko cg ikindi gikoresho atabimennye?	1 Yego<br>0 Oya<br>0.1 Simbizi
LF70 *(required)*	Umwana ashobora kwibanda ku gikorwa kimwe iminota 20 (Urugero: gukina n'inshuti cg kurya)?	1 Yego<br>0 Oya<br>0.1 Simbizi
LF71 *(required)*	Umwana azi nibura amazina y'ibice by'umubiri bibiri (akaboko, amaso cg izuru)?	1 Yego<br>0 Oya<br>0.1 Simbizi
LF72 *(required)*	Iyo weretse umwana ikintu arakimenya (Urugero: igikombe cg inyamaswa), ashobora kumenya uko cyitwa burigihe?	1 Yego<br>0 Oya<br>0.1 Simbizi
LF73 *(required)*	Ese ashobora kuvuga akoresheje amagambo magufi cg imigemo ibiri icyarimwe(Urugero:"mama genda", cg "papa rya")?	1 Yego<br>0 Oya<br>0.1 Simbizi
LF74 *(required)*	Umwana ashobora gukoresha igikoresho (Urugero: inkoni cg ikiyiko) ashaka kugera ku bintu biri kure?	1 Yego<br>0 Oya<br>0.1 Simbizi
LF75 *(required)*	Umwana ashobora kwerekana ko akeneye kujya ku musarani?	1 Yego<br>0 Oya<br>0.1 Simbizi

Field	Question	Answer	
LF76 *(required)*	Ese umwana wawe ashobora kuvuga amagambo atandukanye 10 cg arenga (Urugero: amazina nka " mama" cg "umupira")?	1	Yego
		0	Oya
		0.1	Simbizi
LF77 *(required)*	Ese umwana wawe ashobora kwiyambura umwenda (Urugero: kwikuramo ishati)?	1	Yego
		0	Oya
		0.1	Simbizi
LF78 *(required)*	Ese umwana wawe ashobora ku kubwira niba ananiwe cg ashonje?	1	Yego
		0	Oya
		0.1	Simbizi
LF79 *(required)*	Umwana abasha gusoza igikorwa akunda (Urugero: umukino cg igitabo)?	1	Yego
		0	Oya
		0.1	Simbizi
LF80 *(required)*	Umwana ashobora gukora ibikorwa 2 bitandukanye mu buryo bworoshye (Urugero: nko gusubira mu mukino nyuma)	1	Yego
		0	Oya
		0.1	Simbizi
LF81 *(required)*	Ese umwana ashobora kuririmba indirimbo ngufi cg agasubiramo ibice biyigize yafashe mu mutwe ntawumwibukije?	1	Yego
		0	Oya
		0.1	Simbizi
LF82 *(required)*	Ese umwana ashobora gusimbuka akava k'ubutaka n'amaguru yombi?	1	Yego
		0	Oya
		0.1	Simbizi
LF83 *(required)*	Ese umwana ashobora kuvuga interuro igizwe n'amagambo 3 ari kumwe (Urugero: "ndashaka amazi" cg "inzu ni nini")?	1	Yego
		0	Oya
		0.1	Simbizi
LF84 *(required)*	Umwana ashobora kongorera	1	Yego
		0	Oya
		0.1	Simbizi
LF85 *(required)*	Umwana ashobora gusuhuza abaturanyi cg abandi bantu azi atabibwirijwe (Urugero: kuvuga mwaramutse cg gutanga umukono/akaboko)?	1	Yego
		0	Oya
		0.1	Simbizi
LF86 *(required)*	Umwana ashobora gufungura umupfundikizo w'icupa cg w'ijerekani	1	Yego
		0	Oya
		0.1	Simbizi
LF87 *(required)*	Ese umwana ashobora gukoresha neza amagambo akurikira: "iki" " ikihe", "hehe" cg " inde"?	1	Yego
		0	Oya
		0.1	Simbizi
LF88 *(required)*	Ese umwana ashobora gukoresha neza amagambo akurikira: "Njyewe", " Wowe", "we" (Urugero: " Ngiye kuri butike" cg " arya umuceri")?	1	Yego
		0	Oya
		0.1	Simbizi
LF89 *(required)*	Umwana azi kuvuga neza amagambo menshi akoresha?	1	Yego
		0	Oya
		0.1	Simbizi
LF90 *(required)*	Ese umwana ashobora kubara kugeza ku bintu 5 (Urugero: intoki, abantu)?	1	Yego
		0	Oya
		0.1	Simbizi
LF91 *(required)*	Ese umwana ajya abaza abandi bantu batari ababyeyi be, amenyereye kubona iyo badahari? (Urugero: "umuturanyi arihe?)	1	Yego
		0	Oya
		0.1	Simbizi
LF92 *(required)*	Iyo weretse umwana ibintu bibiri cg abantu, bifite ingano/imibyimba itandukanye ashobora gutandukanya ikinini n'igito?	1	Yego
		0	Oya
		0.1	Simbizi
LF93 *(required)*	Umwana ashobora guhagarara ku kirenge kimwe akamara akanya nta muntu yifashishije cg ikintu?	1	Yego
		0	Oya
		0.1	Simbizi
LF94 *(required)*	Ese umwana ashobora kwerekana nibura ibara rimwe (Urugero: umutuku, ubururu, umuhondo)?	1	Yego
		0	Oya
		0.1	Simbizi
LF95 *(required)*	Umwana akoresha neza kenshi amagambo nka: "vuba", "gufi" "gishyushye", "kibyibushye" cg" "cyiza"	1	Yego
		0	Oya
		0.1	Simbizi
LF96 *(required)*	Ese iyo ukoze ku kintu, umwana wawe ashobora gukoresha neza amagambo “ku”, “mu”, cyangwa “munsi” mu gusobanura aho kiri (urugero,” igikombe kiri ku meza” aho kuba “igikombe kiri mu meza.”)	1	Yego
		0	Oya
		0.1	Simbizi

Field	Question	Answer
LF97 *(required)*	Ese umwana ashobora gusobanura mu magambo akamaro k'intebe n'ibikombe?	1 Yego<br>0 Oya<br>0.1 Simbizi
LF98 *(required)*	Ashoobora kwiyambika imyenda ubwe(Urugero: "kwiyambika ipantalo n'ishati ntabufasha abonye?)	1 Yego<br>0 Oya<br>0.1 Simbizi
LF99 *(required)*	Ese umwana ajya abaza ikibazo akoresheje "kubera iki" (Urugero: "Kubera iki uri muremure?")?	1 Yego<br>0 Oya<br>0.1 Simbizi
LF100 *(required)*	Iyo usabye umwana kuguha ibintu bitatu (Urugero: amabuye, ibishyimbo), aguha umubare wa nyawo?	1 Yego<br>0 Oya<br>0.1 Simbizi
LF101 *(required)*	Umwana iyo arangije gukina asubiza ibikinisho cg ibindi bintu mu mwanya wabyo?	1 Yego<br>0 Oya<br>0.1 Simbizi
LF102 *(required)*	Umwana ajya yitwara mu buryo budasanzwe cg agakora ikintu ahubutse (Urugero: nko kwirukira mu muhanda atarebye?	0 Yego<br>1 Oya<br>0.1 Simbizi
LF103 *(required)*	Umwana ajya azigamira ejo hazaza ibintu nka bonbon cg ibikinisho bishya ?	1 Yego<br>0 Oya<br>0.1 Simbizi
LF104 *(required)*	Umwana ashobora kuvuga ibyo abandi bakunda cg badakunda (Urugero: "mama ntakunda imbuto", "papa akunda umupira w'amaguru")?	1 Yego<br>0 Oya<br>0.1 Simbizi
LF105 *(required)*	Umwana ashobora gufunga cg gufungura ibipesu ntabufasha ahawe?	1 Yego<br>0 Oya<br>0.1 Simbizi
LF106 *(required)*	Umwana azi gukoresha inzagihe neza (Urugero: "ejo azajya kwishuri", " Icyumweru gitaha tuzajya ku isoko")?	1 Yego<br>0 Oya<br>0.1 Simbizi
LF107 *(required)*	Umwana azi gukoresha neza impitagihe (Urugero: " Ejo hashize nakinanye na mugenzi wanjye" cg " mu cyumweru gishize yagiye ku isoko")?	1 Yego<br>0 Oya<br>0.1 Simbizi
LF108 *(required)*	Umwana azi kuvuga izi nyuguti (Urugero: A,B,C,)?	1 Yego<br>0 Oya<br>0.1 Simbizi
LONG FORM: MENTAL HEALTH		
LMH_note	Turacyafite ibindi bibazo bike byo gusubiza. Amabwiriza ni amwe n'ayambere. Mukomeze gusubiza mukoresheje "yego", "oya", cg "simbizi"	
LMH1 *(required)*	Ese umwana arizwa n'ubusa (Urugero: adashonje cg atananiwe)?	0 Yego<br>1 Oya<br>0.1 Simbizi
LMH2 *(required)*	Umwana akunda gutaka umutwe no mu nda kandi ubona afite imbaraga	0 Yego<br>1 Oya<br>0.1 Simbizi
LMH3 *(required)*	Umwana ararakara iyo mutari kumwe	0 Yego<br>1 Oya<br>0.1 Simbizi
LMH4 *(required)*	Umwana agira amasoni, ubwoba, uburakari iyo arikumwe n'abantu atazi, niyo mwaba muri kumwe?	0 Yego<br>1 Oya<br>0.1 Simbizi
LMH5 *(required)*	Ese umwana yizirika k'umurezi we, niyo baba bari ahantu hizewe?	0 Yego<br>1 Oya<br>0.1 Simbizi
LMH6 *(required)*	Umwana ashobora kwicara iyo yabisabwe n'umuntu mukuru (Urugero: akamara iminota 2)?	1 Yego<br>0 Oya<br>0.1 Simbizi
LMH7 *(required)*	Umwana ararira cg araboroga mugihe bamusabye gutegereza ikintu ashaka (Urugero: igikinisho cg ibiryo)?	0 Yego<br>1 Oya<br>0.1 Simbizi
LMH8 *(required)*	Umwana ajya ashota, aruma cg agakubita abandi bana cg abantu bakuru?	0 Yego<br>1 Oya<br>0.1 Simbizi
LMH9 *(required)*	Umwana arigunga cg agira amasoni iyo ageze ahantu atamenyereye?	0 Yego<br>1 Oya<br>0.1 Simbizi

Field	Question	Answer
CHILD DISABILITY		
da_intro	Ndifuza ko twaganira birambuye kuri ({child_name}) n'ubuzima bwe. Ikiganiro kiradutwara iminota mike.	
disability1 *(required)*	Ugereranije n'abandi bana,yaba ({child_name}) yarakererewe bikabije kwicara,cg kugenda?	0 Oya<br>1 Yego<br>99 Simbizi
disability2 *(required)*	Ugereranyije n'abandi bana yaba ({child_name}) afite ibibazo byo kureba/kubona neza byaba kumanywa cg ninjoro?	0 Oya<br>1 Yego<br>99 Simbizi
disability3 *(required)*	Yaba ({child_name}) agaragaraho ibibazo byo kumva (yifashisha utwuma tumufasha kumva cg atumva burundu)	0 Oya<br>1 Yego<br>99 Simbizi
disability4 *(required)*	Iyo ubwiye ({child_name}) kugira icyo akora, yaba yumva ibyo umubwira?	0 Oya<br>1 Yego<br>99 Simbizi
disability5 *(required)*	Yaba ({child_name}) afite ikibazo cyo kugenda,kunyeganyeza ukuboka cg afite intege nke cg ikibazo mu maboko cg amaguru?	0 Oya<br>1 Yego<br>99 Simbizi
disability6 *(required)*	Yaba ({child_name}) rimwe na rimwe aba ameze neza, agahinduka umunyamahame cg agata ubwenge?	0 Oya<br>1 Yego<br>99 Simbizi
disability7 *(required)*	Yaba ({child_name}) yiga gukora ibintu nk'abandi bana bo mu kigero cye?	0 Oya<br>1 Yego<br>99 Simbizi
disability8 *(required)*	Yaba ({child_name}) azi kuvuga, (ashobora kuvuga amagambo yumvikana; ashobora kuvuga amagambo ukamenya icyo ashatse kuvuga)?	0 Oya<br>1 Yego<br>99 Simbizi
disability9 *(required)*	Yaba ({child_name}) avuga mu buryo butandukanye n'ubusanzwe (budasobanutse kuburyo atumvwa n'abantu batari abo mu muryango we wa hafi)?	0 Oya<br>1 Yego<br>99 Simbizi
disability10 *(required)*	Yaba ({child_name}) ashobora kuvuga izina byibuze ry'ikintu kimwe (urugero, inyamaswa, igikinisho, igikombe, ikiyiko)?	0 Oya<br>1 Yego<br>99 Simbizi
disability11 *(required)*	Ugereranyije n'abandi bana bo mukigero cye yaba ({child_name}) agaragaza mu buryo ubw'ari bwo bwose gukererwa ku bwonko, cg bukora buhoro?	0 Oya<br>1 Yego<br>99 Simbizi
Ages and Stages Questionnaire		
ASQ_note	The ASQ is a copyrighted measure available from https://agesandstages.com/	
Check Out		
F_ID_check *(required)*	Andika umubare w'ibanga ugenewe umuryango (Study ID)	
comments	Please enter any comments about the interview that you would like to share	
